# Dynamics of ferrofluidic flow in the Taylor-Couette system with a small aspect ratio

**DOI:** 10.1038/srep40012

**Published:** 2017-01-06

**Authors:** Sebastian Altmeyer, Younghae Do, Ying-Cheng Lai

**Affiliations:** 1 Institute of Science and Technology Austria (IST Austria), 3400 Klosterneuburg, Austria; 2 Department of Mathematics, KNU-Center for Nonlinear Dynamics, Kyungpook National University, Daegu, 41566, Republic of Korea; 3 School of Electrical, Computer, and Energy Engineering, Arizona State University, Tempe, Arizona 85287, USA

## Abstract

We investigate fundamental nonlinear dynamics of ferrofluidic Taylor-Couette flow - flow confined be-tween two concentric independently rotating cylinders - consider small aspect ratio by solving the ferro-hydrodynamical equations, carrying out systematic bifurcation analysis. Without magnetic field, we find steady flow patterns, previously observed with a simple fluid, such as those containing *normal* one- or two vortex cells, as well as *anomalous* one-cell and twin-cell flow states. However, when a *symmetry-breaking* transverse magnetic field is present, all flow states exhibit stimulated, finite two-fold mode. Various bifurcations between steady and unsteady states can occur, corresponding to the transitions between the two-cell and one-cell states. While unsteady, *axially oscillating* flow states can arise, we also detect the emergence of new unsteady flow states. In particular, we uncover two new states: one contains only the *azimuthally oscillating* solution in the configuration of the twin-cell flow state, and an-other a *rotating* flow state. Topologically, these flow states are a limit cycle and a quasiperiodic solution on a two-torus, respectively. Emergence of new flow states in addition to observed ones with classical fluid, indicates that richer but potentially more controllable dynamics in ferrofluidic flows, as such flow states depend on the external magnetic field.

The flow between two concentric differentially rotating cylinders, the Taylor-Couette system (TCS), has played a central role in understanding the various hydrodynamic stabilities^1^,^2^ TCS has been a paradigm to investigate many fundamental nonlinear dynamical phenomena in fluid flows. The simplicity of the geometry of the system allows for well-controlled experimental studies. The vast literature in this area has been built on the TCS with a simple fluid. (For convenience, in this paper we call TCS with a simple fluid the *‘classical TCS’*). Recently there has been an increasing amount of interest in the flow dynamics of the TCS with a complex fluid^3^,^4^,^5^,^6^,^7^,^8^,^9^. A representative type of complex fluids is ferrofluids^10^, which are manufactured fluids consisting of dispersion of magnetized nanoparticles in a liquid carrier. A ferrofluid can be stabilized against agglomeration through the addition of a surfactant monolayer onto the particles. In the absence of any magnetic field, the nanoparticles are randomly orientated so that the fluid has zero net magnetization. In this case, the nanoparticles alter little the viscosity and the density of the fluid. Thus, in the absence of any external field a ferrofluid behaves as a simple (classical) fluid. However, when a magnetic field of sufficient strength is applied, the hydrodynamical properties of the fluid, such as the viscosity, can be changed dramatically^11^,^12^ and the dynamics can be drastically altered. Studies indicated that, under a symmetry-breaking transverse magnetic field, all flow states in the TCS become intrinsically *three-dimensional*^3^,^5^,^7^. As such, a magnetic field can have a significant influence on the hydrodynamical stability and the underlying symmetries of the flow states through, e.g., certain induced azimuthal modes^7^. Aside this a change in the magnetic field strength can also induce turbulence^8^. Ferrofluidic flows have wide applications, ranging from gaining insights into the fundamentals of geophysical flows through laboratory experiments^13^,^14^ to the development of microfluidic devices and computer hard drives. For example, a recent study demonstrated that ferrofluidic flows in the TCS can reverse their directions of rotation spontaneously^9^, which has implications to the phenomenon of geomagnetic reversal^14^,^15^,^16^,^17^,^18^,^19^,^20^,^21^.

Our study of the ferrofluidic flow states in the TCS with a small aspect ratio was motivated by the following considerations. Previous numerical and experimental works demonstrated that the effects of end walls are *not* negligible^22^,^23^,^24^,^25^ even in the large aspect ratio TCS. The walls can thus have a *significant* effect on the flow dynamics. For the classical TCS or for TCS with a ferrofluid but without any magnetic field, for small aspect ratio (e.g., Γ ≈ 1) the flow dynamics is dominated by the competition between normal and anomalous flow states, leading to rich dynamical behaviors^26^,^27^,^28^,^29^,^30^. Here the term “*normal*” (“*anomalous*”) is referred to as a flow state with vortex cells that give an inward (outward) flow near each lid in the radial direction. For systems of a small height different flows patterns with one (*one-cell flow state*) or two (*two-cell flow state*) Taylor vortex cells were detected^31^,^32^. A plausible mechanism for the emergence of the flow states is that the vortex cells, independent of the normal or anomalous nature of the flow, divide the flow in the axial direction. In addition, flows with two identical cells, the so called “twin-cell” flows, were observed^33^, in which the two vortex cells divide the flow in the radial instead of the common axial direction. That is, both cells touch the top and the bottom lids. While most flow states are steady, an unsteady and axially oscillatory flow state was also experimentally detected^34^ and numerically demonstrated^33^. An alternative type of unsteady flow states with more complex dynamics^35^ was observed in the TCS with an extraordinarily small aspect ratio (e.g., Γ = 0.5), where the flow can change among two, three, and four cells in a radially separating configuration over one period. To summarize briefly, existing works on the classical TCS demonstrated that complex oscillatory flow patterns can arise when the aspect ratio of the system is reduced. An open issue is what types of dynamical behaviors can arise in the flow patterns in the ferrofluidic TCS, subject to a magnetic field.

In this paper, we report the results from a systematic computational study of the ferrofluidic flow dynamics in the TCS with a small aspect ratio, i.e., on the order of unity, which we choose as a bifurcation parameter (The radius ratio of the cylinders [inner cylinder radius/outer cylinder radius] is fixed to 0.5). Another bifurcation parameter is the Reynolds number (*Re*_2_ = *ω*_2_*r*_2_*d/ν* see Methods) of the outer cylinder. Specifically, we set the rotation speed of the inner cylinder so as to fix its Reynolds number at *Re*_1_ = 250, and vary the rotation speed of the outer cylinder. Both end walls confining the TCS are stationary. To distinguish from the dynamics of a simple fluid, we apply a symmetry breaking, transverse magnetic field. The main results can be stated as follows. We find that all flow states exhibit a general feature: they contain a stimulated two-cell mode^5^,^25^,^36^. As the aspect ratio is changed, various bifurcations between steady and unsteady flow states can occur, corresponding to the transitions between the two-cell and one-cell states. While unsteady, axially oscillating flow states similar to those in a simple fluid can occur, novel unsteady flow states that are not found in the classical TCS can arise. In particular, we uncover two new states: one that contains only the *azimuthally* oscillating solution in the configuration of the twin-cell flow state, and another a rotating flow state, which correspond topologically to limit cycle and quasiperiodic solution on a two-torus, respectively. Due to the sequence of bifurcations following a symmetry breaking bifurcation, the one-cell and twin-cell flow states are symmetrically related. We also uncover various regions of bistability with the coexistence of one- and two-cell flow states. The emergence of the novel flow states in addition to those occurring typically in the classical TCS suggests that the ferrofluidic TCS can exhibit richer dynamics that are potentially more controllable due to their dependence on an additional experimentally adjustable parameter: the magnetic field strength.

## Results

### Nomenclature

We focus on the flow states in the small aspect-ratio TCS. A common feature shared by all flow states is that the axisymmetric Fourier mode associated with the azimuthal wavenumber *m* = 0 (see Methods) is dominant so that the flow states correspond to *toroidally closed* solutions. Note that ferrofluidic flows dominated by an azimuthally modulated *m* = 0 mode differ from the classical wavy vortex flow solutions in the absence of any magnetic field^37^,^38^,^39^,^40^,^41^, which are time-periodic, *rotating* states that do *not* propagate axially. In the presence of a transverse magnetic field, all the flow states are fundamentally three dimensional with a stimulated *m* = 2 mode, leading to steady (*non-rotating*) wavy vortex flows^3^,^5^,^7^. Rotating flows with a finite *m* = 1 mode can also arise, so do unsteady (oscillatory) flow solutions. A key indicator differentiating various flow states is the number of vortex cells present in the annulus, i.e., in the (*r, z*) plane. To take into account all the differences, we use the notation 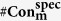 defined in Table 1 to distinguish the different flow patterns. For example, the notion 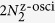 stands for an unsteady, axially oscillating [z-osci] two-cell^2^ flow state in the normal configuration [N] with a stimulated *m* = 2 mode^2^. It is worth mentioning that all calculated wavy flows are *stable*. However, for the parameter regimes considered the Taylor-vortex flow (TVF) solutions are unstable. The magnetic field strength can be characterized by the Niklas parameter (see Methods). In the present work we consider a transverse field with fixed parameter *s*_*x*_ = 0.6. The velocity and vorticity fields are ***u*** = (*u, v, w*) and ∇ × ***u*** = (*ξ, η, ζ*), respectively.

### Parameter space and quantities

**Figure 1:**
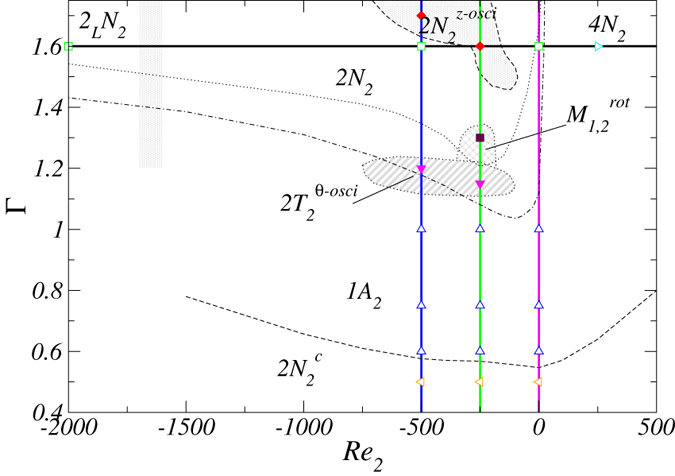
Cases of simulations and analysis carried out in the parameter space (Γ, *Re*_2_). We focus on the variation in the Reynolds number of the outer cylinder, 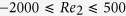 for fixed aspect ratio Γ = 1.6, or on variations in the aspect ratio for fixed *Re*_2_ = −500,−250 and 0, as indicated by the straight and horizontal lines, respectively. Different symbols highlight the parameters for which the flow states are studied in greater detail. The dashed and dotted lines specify the parameter values for which only a qualitative analysis of the flow states is carried out in terms of their existence and stability. Within the region between the dotted and dashed-dotted curves, one-cell and two-cell flow states exist and are bistable.

Figure 1 provides an overview of the structure of the parameter space (Γ, *Re*_2_) investigated in this paper. Simulations for the parameters specified by the solid horizontal and the vertical lines are carried out and different symbols highlight the parameter values for which flow states are studied in great detail. For the dashed and dotted lines, the parameters are chosen according to the steps Δ*Re*_2_ = 50 and ΔΓ = 0.02.

As a global measure to characterize the flow, we use the modal kinetic energy defined as


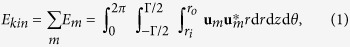


where **u**_*m*_ (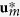) is the *m*-th (complex conjugate) Fourier mode of the velocity field, *E*_*kin*_ is constant (non-constant) for a steady (an unsteady) solution. For a diagnostic purpose, we consider the time-averaged (over one period) quantity, 
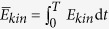
. In addition to the global measure, we also use the azimuthal vorticity on the inner cylinder at two points symmetrically displaced about the mid-plane, *η*_±_ = (*r*_*i*_, 0, ±Γ/4, *t*), as a local measure to characterize the flow states (see Methods). The unsteady, *oscillating* flow states in the axial and the azimuthal directions are key dynamical states of the underlying TCS system with a complex fluid. In order to obtain the axial and/or azimuthal frequencies, we first conduct visualization of the full flow state to decide if the flow structure is oscillating in the axial or the azimuthal direction, or even rotating as a whole. In the case of oscillating states (axial or azimuthal), the full flow fields are identical after a period time *τ*. The inverse of this period time defines the frequency *ω*. We then calculate the power spectral densities (PSDs) of the global quantity *E*_*kin*_ as well the local quantities *η*_±_, taking into account the system symmetries. These two steps, together with the knowledge of the spatiotemporal behavior of the flow structure, give the frequency of the flow state. A more detailed description of our classication scheme for axially oscillating, azimuthally oscillating, or rotating flow states is presented in Supplementary Materials.

### Bifurcation with *Re*_*2*_

**Figure 2:**
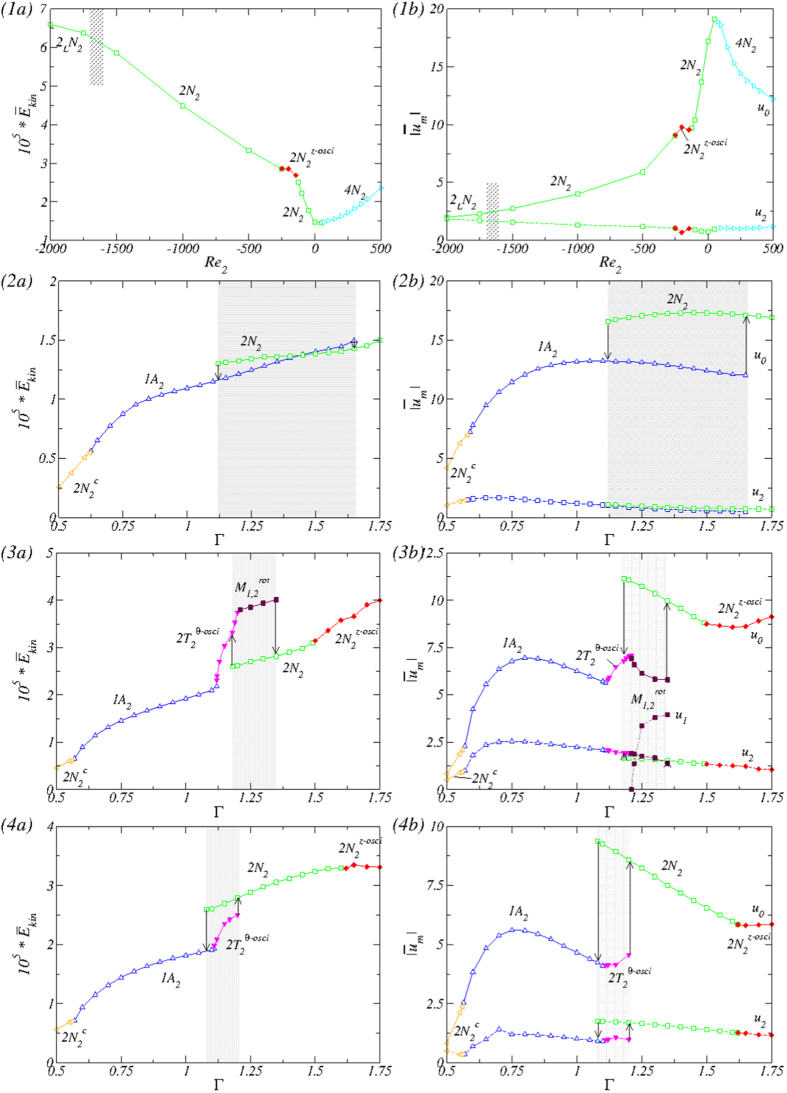
Bifurcation scenarios. Bifurcation scenarios with (1) the Reynolds number *Re*_2_ of the outer cylinder at fixed Γ = 1.6 and bifurcation with the aspect ratio Γ for (2) *Re*_2_ = 0, (3) *Re*_2_ = −250 and (4) *Re*_2_ = −500, respectively. Shown are (**a**) the total (time-averaged for oscillatory flows) modal kinetic energy 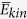 and (**b**) the corresponding dominant (time-averaged) amplitudes 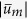 of the radial velocity field at mid-gap contributed by the axisymmetric mode [*u*_0_, solid line in (**b**)] and the *m* = 2 mode [*u*_2_, dashed line in (**b**)]. Full (empty) symbols represent the time-dependent (time-independent) flows. Different flow structures are labeled. The highlighted gray areas indicate the region of coexistence of distinct flow states: *Re*_2_ = −0:1.119 ≤ Γ ≤ 1.657; *Re*_2_ = −250:1.18 ≤ Γ ≤ 1.34; *Re*_2_ = −500:1.108 ≤ Γ ≤ 1.21.

To detect and understand the emergence of novel flow patterns in ferrofluidic TCS with a small aspect ratio, we start from a moderate value so that the conventional two-cell flow state occurs^26^,^27^,^30^,^42^. To be concrete, we set Γ = 1.6 and take *Re*_2_ as a bifurcation parameter. Figure 2(1) shows the total modal kinetic energy 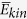 (*a*) together with the axisymmetric [*u*_0_, the solid line in (*b*)] and two-fold symmetric [*u*_2_, dashed line in (*b*)] mode amplitudes versus *Re*_2_. Note that the two-fold symmetric mode is intrinsically stimulated when a transverse magnetic field is present^5^,^7^,^36^,^43^.

#### The case of *Re*_2_ = 0

**Figure 3:**
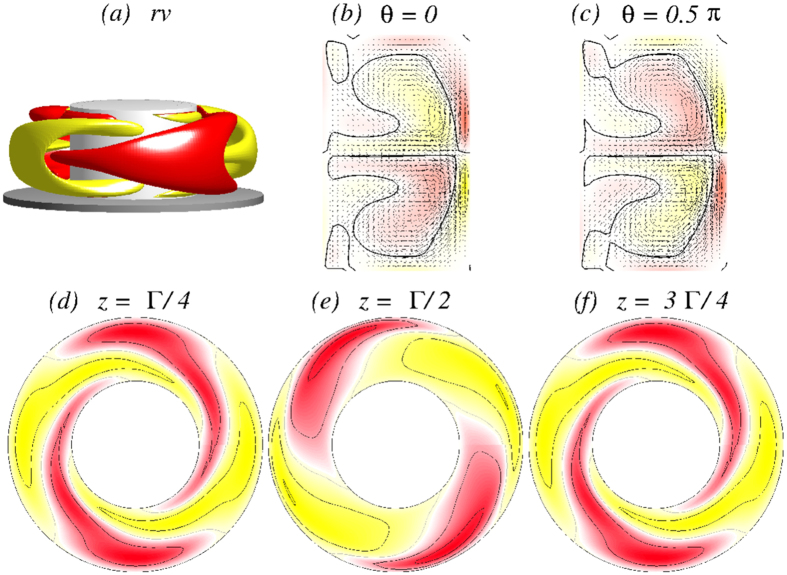
Flow visualization of the 2*N*_2_ state. Flow visualization of 2*N*_2_ for Γ = 1.6 and *Re*_2_ = 0: (**a**) isosurface of *rv* (isolevel shown at *rv* = ±5) and the corresponding vector plot [*u(r, z*), *w(r, z*)] of the radial and axial velocity components (including the azimuthal vorticity *η(r, θ*)) for (**b**) *θ* = 0 and (**c**) *θ* = *π*/2. (**d–f**) The azimuthal velocity *v(r, θ*) in three different planes: *z* = Γ/4, *z* = Γ/2, and *z* = 3Γ/4, respectively. The same legends are used for visualizing all the time independent flows in the paper.

When the outer cylinder is at rest (*Re*_2_ = 0), a two-cell flow state 2*N*_2_ is developed, as shown in Fig. 3. Due to the two-fold symmetry, this state differs little from the classical two-cell state in the absence of the magnetic field. The state possesses both the 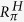 and 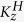 symmetries^7^ (see Methods). The isosurface plot for *rv* = ±5 and the cross-sections in the (*r, z*) plane indicate the two-fold rotational symmetry 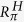 of the 2*N*_2_ state: *η(r, θ* = 0, *z*) = −*η(r, θ* = *π*/2, *z*), whereas the horizontal cuts exhibit the 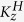 invariance.

#### The case of *Re*_2_ > 0

**Figure 4:**
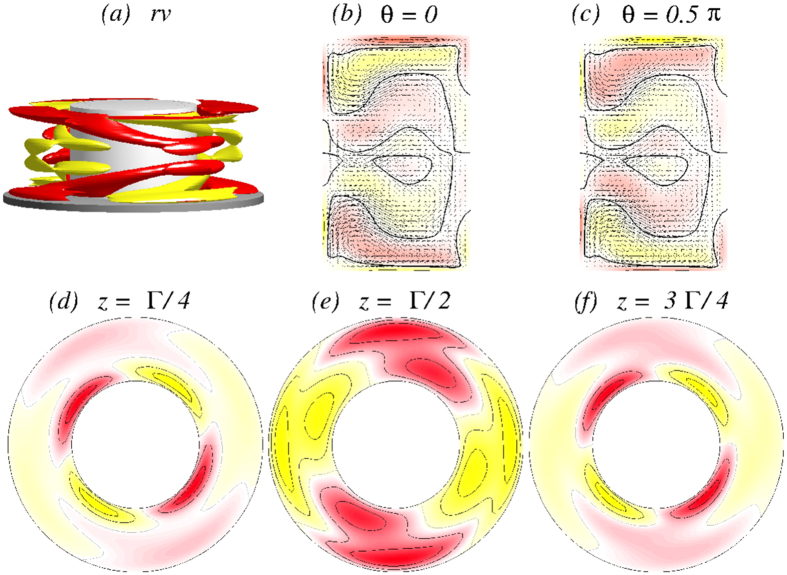
Visualization of flow state 4*N*_2_. The 4*N*_2_ flow state for Γ = 1.6 and *Re*_2_ = 250. The legends are the same as in Fig. 3. The isosurface for *rv* = ±7 is shown in (**a**).

Increasing the value of *Re*_2_ from zero so that the cylinders rotate in the same direction, the flow starts to develop two additional symmetrical vortex cells. These cells appear for *Re*_2_ ≈ 27, which are initially located in the corners near the inner cylinder and the lids. As a result, a four-cell flow state emerges, denoted as 4*N*_2_, which has the same symmetries (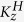, 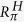) as the two-cell state 2*N*_2_ that it emerges from. An example of 4*N*_2_ for co-rotating cylinders at exactly the same speed is presented in Fig. 4. As *Re*_2_ is increased, the original two-cells are pulled closer towards the outer cylinder, giving more space for the two additional cells that extend into the interior of the bulk. This effect becomes continuously stronger for larger values of *Re*_2_. For the parameter range investigated, i.e., 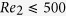, the flow state remains qualitatively the same as that shown in Fig. 4. Increasing *Re*_2_ also leads the kinetic energy 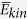 to increase continuously. During this process the contribution to the energy from the dominant axisymmetric *m* = 0 mode (*u*_0_) decreases while that from the *m* = 2 mode (*u*_2_) increases slightly, as shown in Fig. 2(1).

#### The case of *Re*_2_ < 0

As *Re*_2_ is decreased from zero, the two cylinders become counter-rotating. Initially the flow state 2*N*_2_ remains unchanged. As *Re*_2_ is decreased through a critical value of about −180, the state 2*N*_2_ loses its stability via a supercritical symmetry-breaking Hopf bifurcation at which the mid-plane reflection symmetry 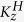, together with a reversal of the magnetic field (cf., Eq. (10) and ref. 36 and Methods), is broken and is replaced by a spatial temporal symmetry *S*^*H*^ consisting of the mid-plane reflection 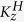 in combination with a half-period time evolution Φ_*τ*/2_. The physical manifestation of this symmetry breaking phenomenon can be seen by noting that the two vortex cells now *oscillate axially* about the mid-plane. However, the new flow state 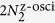 is *not* a rotating state (which is the most typical case in TCS when the flow becomes time-dependent ^?, ?, ?^).

**Figure 5:**
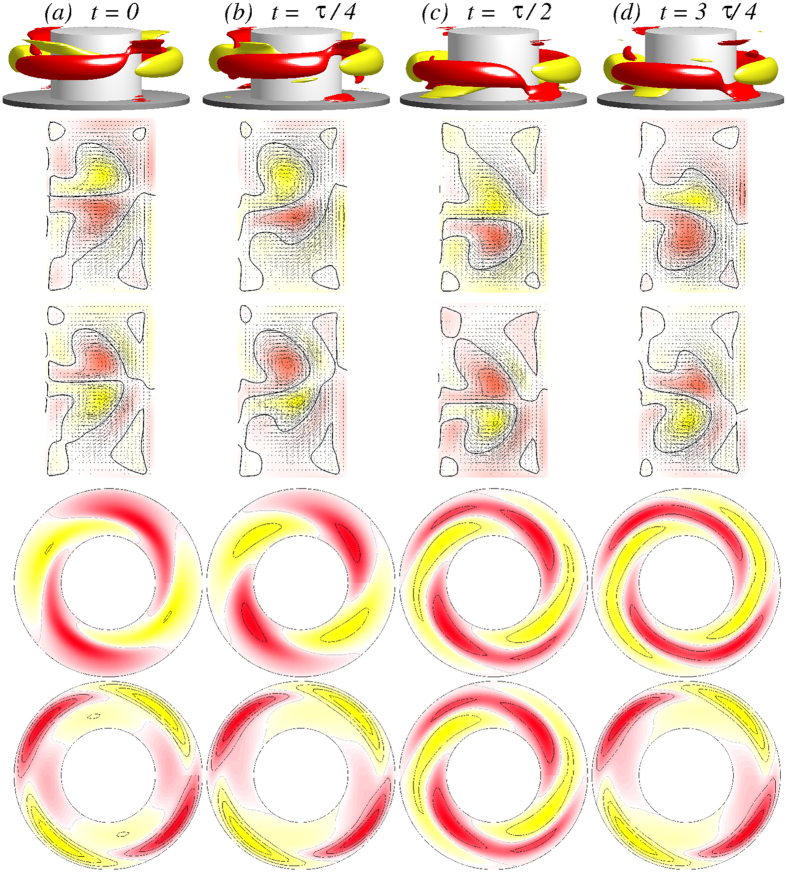
Visualization of the axially oscillating flow state 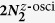. The first row shows, for Γ = 1.6 and *Re*_2_ = −250, the isosurfaces of *rv* (isolevel shown at *rv* = ±15) over one axially oscillating period (*τ*_*z*_ ≈ 0.1635) at instants of time *t* as indicated. The second and third rows show the corresponding vector plots [*u(r, z*), *w(r, z*)] of the radial and axial velocity components in the planes defined by *θ* = 0 and *θ* = *π*/2, respectively, where the color-coded azimuthal vorticity field *η* is also shown. The fourth and fifth rows represent the azimuthal velocity *v(r, θ*) in the axial planes *z* = Γ/4 and *z* = Γ/2, respectively. Red (dark gray) and yellow (light gray) colors correspond to positive and negative values, respectively, with zero specified as white. See also movie file movieA1.avi, movieA2.avi, movieA3.avi and movieA4.avi in Supplementary Materials (SMs) [The same axially oscillating flow state 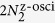 for different parameters is preented in SMs: movieE1.avi, movieE2.avi and movieE3.avi]. The same legends for flow visualization are used for all subsequent unsteady flows.

In order to get more insight the flow dynamics Fig. 5 presents four snapshots of the axially oscillating flow state 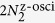 [see also SMs: movieA1.avi movieA2.avi, movieA3.avi and movieA4.avi]. Shown are the angular momentum *rv*, vertical cross-section plots of *η(r, θ* = 0[*π*/2], *z*), and horizontal cross-section plots of *v(r, θ, z* = 1/4[1/2]Γ) over one period (*τ*_*z*_ ≈ 0.1635) illustrating the axial oscillation of the vortex cells. The figure also demonstrates the half-period flip symmetry *S*^*H*^, where 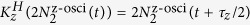. The effect of *S*^*H*^ on the velocity field is


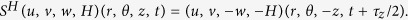


**Figure 6:**
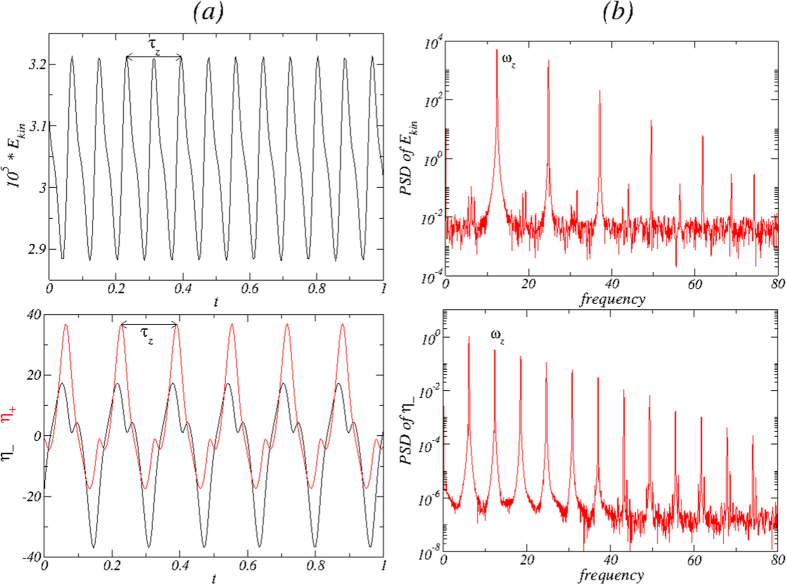
Time series and PSD for the axially oscillating flow state 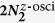. For Γ = 1.6 and *Re*_2_ = −250, (**a**) time series of *E*_*kin*_, *η*_+_ [red (gray)], *η*_−_ (black). (**b**) The corresponding power spectral densities (PSDs) of the 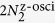 state. The period of axial oscillation is *τ*_*z*_ ≈ 0.1635 with the corresponding frequency *ω*_*z*_ ≈ 12.232. Note that the PSD of the local quantity *η*_−_ has a peak at about half of this frequency, indicating the half-period flip symmetry *S*^*H*^ of the solution.

Topologically speaking the axially oscillating flow state 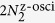 is a limit cycle solution oscillating with the frequency *ω*_*z*_ in the axial direction (cf., PSDs in Fig. 6). The state is thus qualitatively equivalent to the axially oscillating flow state in the classical TCS, which was first detected by Buzug *et al*.^34^. The difference is that, in our ferrofluidic TCS, there is a finite contribution from the *m* = 2 modes. The limit cycle characteristic of 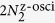 results in closed curves in the phase-space plot [see also SMs Fig. 9].

Figure 6 shows the time series of the modal kinetic energy *E*_*kin*_ and *η*_ ± _together with its corresponding power spectral densities (PSDs) for the 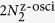 state for Γ = 1.6 and *Re*_2_ = −250. Note that *τ*_*z*_ is twice the period of the time series of *E*_*kin*_ [cf., Fig. 6(*a*)], due to the fact that the 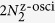 state is half-period flip invariant and so 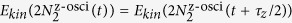, whereas if 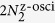 is *τ*_*z*_ periodic, we have 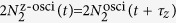. The half-period flip symmetry is visible in the time series of *η*_+_ and *η*_−_.

Further decreasing *Re*_2_, the flow loses its time dependence again (through a similar symmetry breaking Hopf bifurcation but in the reverse direction). For *Re*_2_ ≈ −280, the steady 2*N*_2_ flow state emerges again. Due to the stronger counter rotation of the two cylinders, the vortex cells are slightly shifted towards the inner cylinder wall but remain qualitatively the same, as shown for the case *Re*_2_ = 0 in Fig. 3.

As *Re*_2_ is decreased continuously, the solution remains topologically identical to a two-cell flow state. For *Re* about −1680, the flow undergoes a smooth transition in which the vortex centers are pushed towards the inner cylinder and the cells become elongated in the axial direction. A slightly elongated two-cell flow state 2_*L*_*N*_2_ is shown in SMs in Fig. 1. Except for the small parameter regime in which 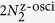 exists, the kinetic energy 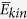 increases continuously with *Re*_2_ (cf., Fig. 2(1)), where the contribution *u*_0_ from the dominant mode *m* = 0 decreases but that from the *m* = 2 mode, *u*_2_, increases. This is confirmed by the fact that stronger counter-rotation flows favor higher azimuthal modes.

### Bifurcation with the aspect ratio Γ

We now fix the value of *Re*_2_ (at 0, −250 and −500), and investigate the bifurcation of the flow state with the aspect ratio Γ, respectively. Note that, in the classical TCS, there can be a transition in the flow between two-cell and one-cell states as Γ is varied. We aim to uncover the similarity and difference in the bifurcations in the ferrofluidic TCS.

#### The case of *Re*_2_ = 0

**Figure 7:**
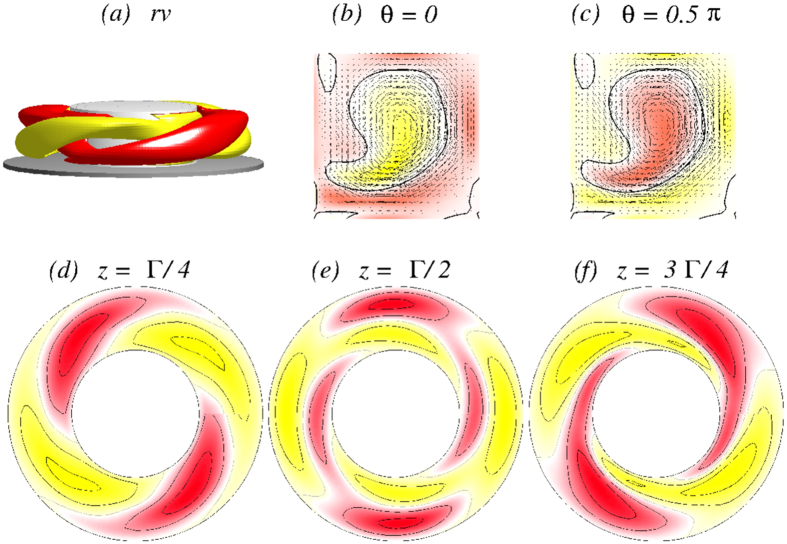
Visualization of flow state 1*A*_2_ for Γ = 1.0 and *Re*_2_ = 0, where panel (a) shows the isosurface for *rv* = ±7. The legends are the same as in Fig. 3.

Figure 2(2) shows, for *Re*_2_ = 0, the variation with Γ of the modal kinetic energy 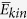 and the dominant amplitude 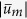 associated with the flow. Increasing Γ from 1.6 (cf., Fig. 2(1) and 3), the flow state 2*N*_2_ remains unchanged and stable until Γ = 1.75. Decreasing Γ from 1.6, the same state holds (cf., Fig. 2(2)) until when Γ ≈ 1.12, where the 2*N*_2_ state loses its stability and becomes a transient. The final flow state has only *one* dominant vortex cell, i.e., the one-cell flow state 1*A*_2_. Figure 7 illustrates the 1*A*_2_ state for Γ = 1.0, where the single vortex nature is apparent. While 1*A*_2_ itself is not 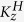 symmetric, there is a coexisting, symmetry possessing flow state 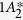 that can be obtained by applying the symmetry operation 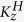: 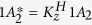. (See below for a detailed explanation). Representative isosurfaces of *rv* are shown in Fig. 7), where a twisted vortex structure with a two-fold symmetry due to the magnetic field can be identified.

**Figure 8:**
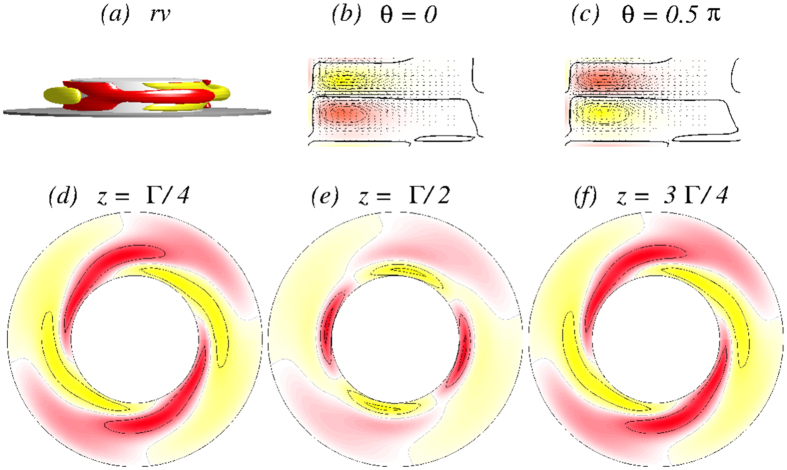
Visualization of flow state 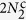 for Γ = 0.5 and *Re*_2_ = 0, where (**a**) shows the isosurface for *rv* = ±5. The legends are the same as in Fig. 3. Note that this flow state was first reported in the classical TCS by Pfister *et al*.^34^, who described it as a two-cell state with two compressed vortices near the inner cylinder.

**Figure 9:**
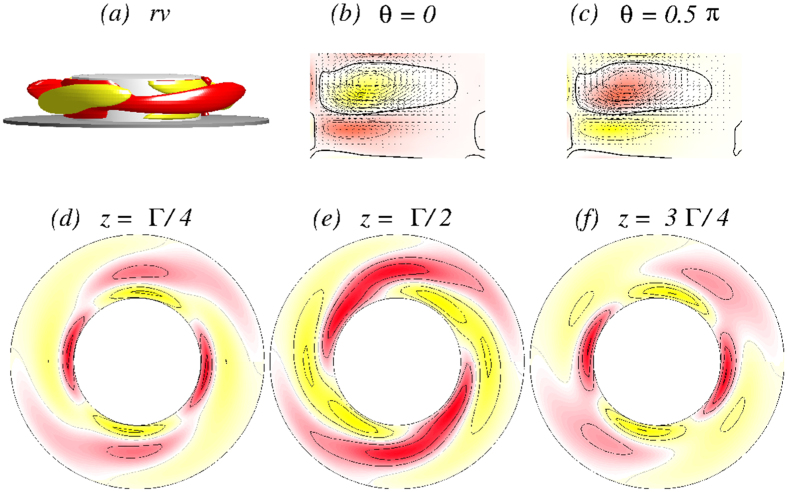
Visualization of flow state 1*A*_2_ for Γ = 0.6 and *Re*_2_ = 0, where (**a**) shows the isosurface for *rv* = ±5. The legends are the same as in Fig. 3.

For clarity and simplicity we now describe the bifurcation sequence and the evolution of the flow state as Γ is increased from the relatively small value of 0.5. For 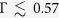, we find the two-cell flow state 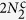 (cf., Fig. 8). Similar to the flow state 2*N*_2_, also 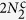 consists of two vortex cells but with the difference that the cells are compressed near the inner cylinder, which results in a wide (outer) region in which the annulus is essentially vortex free. Such states were first reported by Pfister *et al*.^34^ for the classical TCS. The flow state 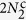 for Γ = 0.5, shown in Fig. 8 which differs from the classical one in only one aspect: a two-fold symmetry induced by the magnetic field^7^. Increasing Γ further the state 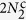 undergoes a symmetry breaking pitchfork bifurcation at Γ ≈ 0.57, where the 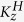 symmetry is broken and one of the vortex cells grows at the cost of the other. As a result, two symmetry related one-cell flow states emerge: 1*A*_2_ and 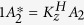. For convenience, from here on we denote them as a one-cell flow states 1*A*_2_ (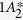), even when one of the vortex cells is slightly smaller than the other. The main dynamics is essentially dominated by the large one. That is, when 1*A*_2_ is mentioned, the coexistence of the 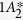 state is implied. An example for 1*A*_2_ after the symmetry breaking bifurcation is shown in Fig. 9 for Γ = 0.6, where it can be seen that the top vortex cell has started to grow (from the bifurcation point) at the cost of the bottom cell. On the *rv* isosurfaces, the formation of the twisted vortex shape can be seen, as a result of the transverse magnetic field, which is typical for 1*A*_2_ (cf., Fig. 7).

**Figure 10:**
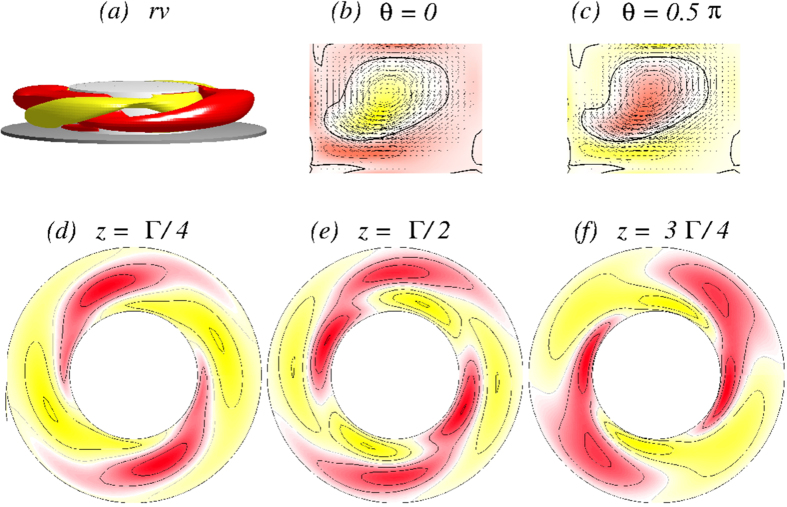
Visualization of flow state 1*A*_2_ for Γ = 0.75 and *Re*_2_ = 0, where (**a**) shows the isosurface for *rv* = ±5. Legends are the same as in Fig. 3.

As Γ is increased further, one of the vortex cells continuously grows in size to occupy more of the interior region of the bulk, while the other becomes increasingly compressed, which can be seen for the 1*A*_2_ state in Fig. 10 for Γ = 0.75, where the dominance of one vortex cell is apparent. In fact, the flow state 1*A*_2_ exists in a wide range of Γ values until it finally loses stability at Γ ≈ 1.66 and becomes a transient to the two-cell flow state 2*N*_2_ that exists for even larger values of Γ (e.g., even for Γ = 1.75 - the largest value of the aspect ratio studied in this paper). These results indicate that, in the parameter interval 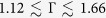, there are two bistable coexisting flow states: a two-cell state 2*N*_2_ and a one-cell state 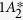. Both, 2*N*_2_ and 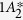 are living on two different *not* connected solution branches.

#### The case of *Re*_2_ = −250

We now study the case of counter-rotating cylinders with *Re*_2_ < 0. The corresponding bifurcation scenario is illustrated in Fig. 2(3). As for *Re*_2_ = −250 and Γ = 1.6 (cf. Fig. 2(1,2)), there is an unsteady, axially oscillating flow state 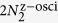, which serves as the baseline for our bifurcation analysis. Increasing Γ from 1.6, this flow state is stable and continues to exist until for Γ = 1.75, whereas if Γ is decreased from 1.6, the state loses its time dependence through a symmetry-breaking, backward Hopf bifurcation for Γ ≈ 1.48, leading again to the flow state 2*N*_2_ (cf., Fig. 3). This is the same bifurcation scenario as described for constant Γ while varying *Re*_2_ (cf., Fig. 2(1)). Upon further decrease in Γ, the 2*N*_2_ state remains stable until for Γ ≈ 1.08 when it loses stability and simultaneously, a single cell state 1*A*_2_ (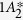) emerges (as described for *Re*_2_ = 0).

Starting again with a small aspect ratio and increasing Γ, the bifurcation sequence is the same as for *Re*_2_ = 0. In particular, for Γ = 0.5 the flow state 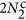 is present, which as Γ is increased undergoes a symmetry breaking pitchfork bifurcation to the symmetry related one-cell flow state 1*A*_2_ (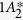). The evolution of 1*A*_2_ as Γ is increased toward unity is qualitatively the same as for the case of *Re*_2_ = 0 [see Fig. 2 in Supplementary Materials], with the small difference being that the vortices have slightly moved towards the inner cylinder due to the counter rotation.

**Figure 11:**
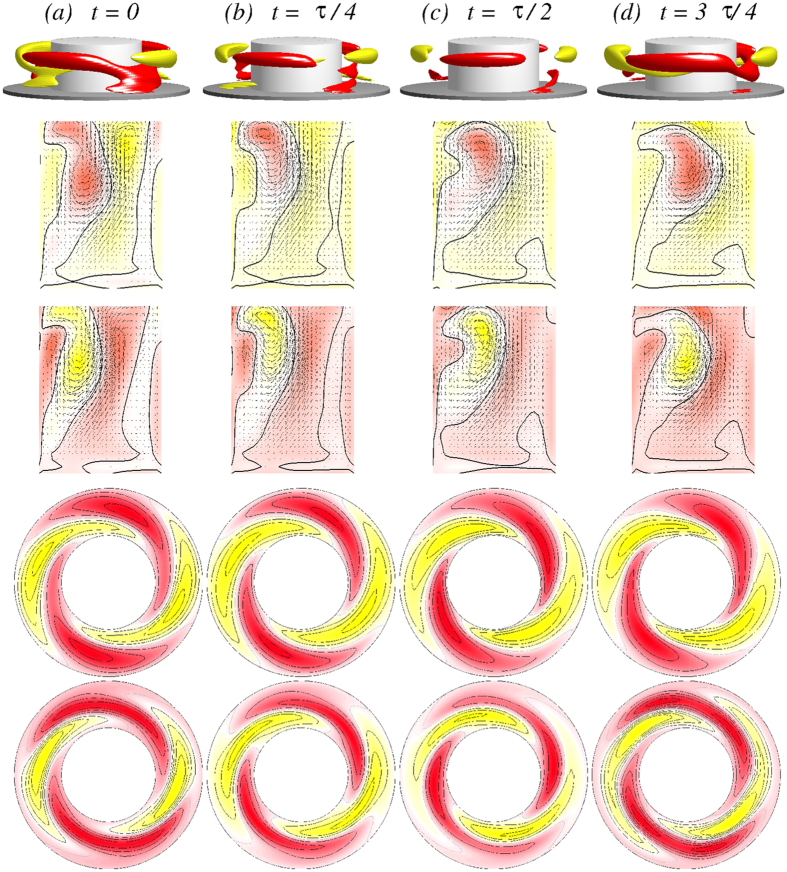
Visualization of the azimuthally oscillating twin-cell flow state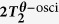 for Γ = 1.15, as in Fig. 5. The top row shows the isosurfaces for *rv* =  ± 25. The oscillation period is *τ*_*θ*_ ≈ 0.0954. See movie files movieB1.avi, movieB2.avi, movieB3.avi and movieB4.avi and movieB4.avi in SMs.

With further increase in Γ the steady one-cell flow state 1*A*_2_ undergoes a Hopf bifurcation, at which an unsteady two-cell flow state emerges with frequency *ω*_*θ*_. For Γ ≈ 1.12, the flow starts to oscillate in the *azimuthal* direction with the frequency *ω*_*θ*_. A difference from the case of axially oscillating flow 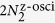 (cf., Figs 5 and 6) is that the new oscillating flow state 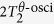 has the characteristics of a *twin-cell* flow state, meaning that the vortex cells are arranged side by side and they both touch the top and the bottom lids, as shown in Fig. 11, instead of being on top of each other. Topologically this flow corresponds to a limit cycle solution, similar to the 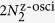 state.

**Figure 12:**
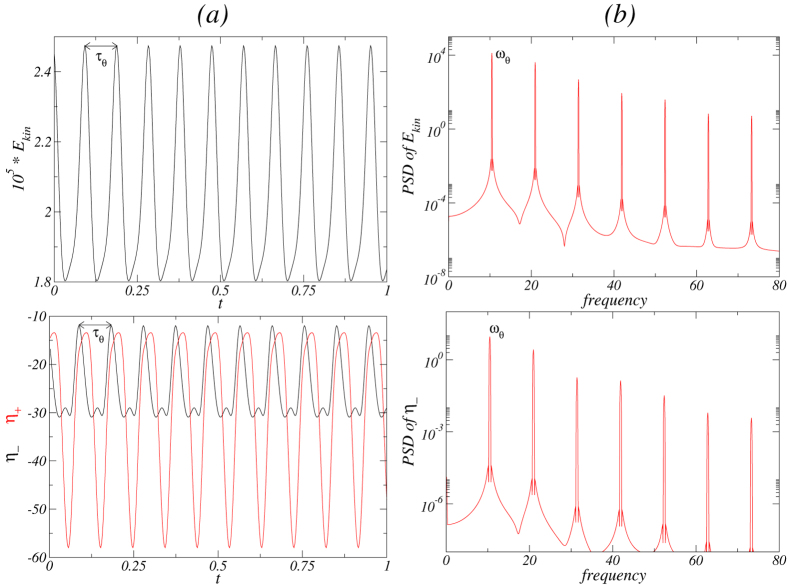
Time series and PSD of the azimuthally oscillating flow state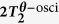. For Γ = 1.15 and *Re*_2_ = −250, (**a**) time series of *E*_*kin*_, *η*_+_ [red (gray)], *η*_−_ (black), and (**b**) the corresponding PSDs for 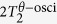. The period of the azimuthal oscillation is *τ*_*θ*_ ≈ 0.0954 with the corresponding frequency *ω*_*θ*_ ≈ 10.482.

Figure 11 demonstrates the 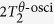 flow state for Γ = 1.15 [see also Supplementary Materials: movieB1.avi, movieB2.avi, movieB3.avi and movieB4.avi]. There are four snapshots of the angular momentum *rv*, vertical cross-section plots of *η(r,θ* = 0[*π*/2], *z*), and horizontal cross-section plots of *v(r, θ, z* = 1/4[1/2]Γ) over one oscillating period *τ*_*θ*_ ≈ 0.0954. Similar to the 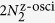 state, 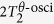 oscillates but in the *azimuthal* direction. Moreover the 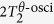 state has no apparent symmetries (cf., Fig. 11), in particular no shift-reflect symmetry *S*^*H*^ as 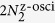 has. The broken symmetry can also be seen in the corresponding time series of *η*_+_ and *η*_−_ in Fig. 12. Note that here the flow state 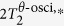 coexists, which evolves in the same way from the symmetry related flow state 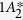 (instead of 1*A*_2_) with increasing Γ. Analogous to the case of one-cell flow state, the symmetry related flow state 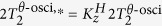 is characterized by a reflection invariance at the mid-height.

**Figure 13:**
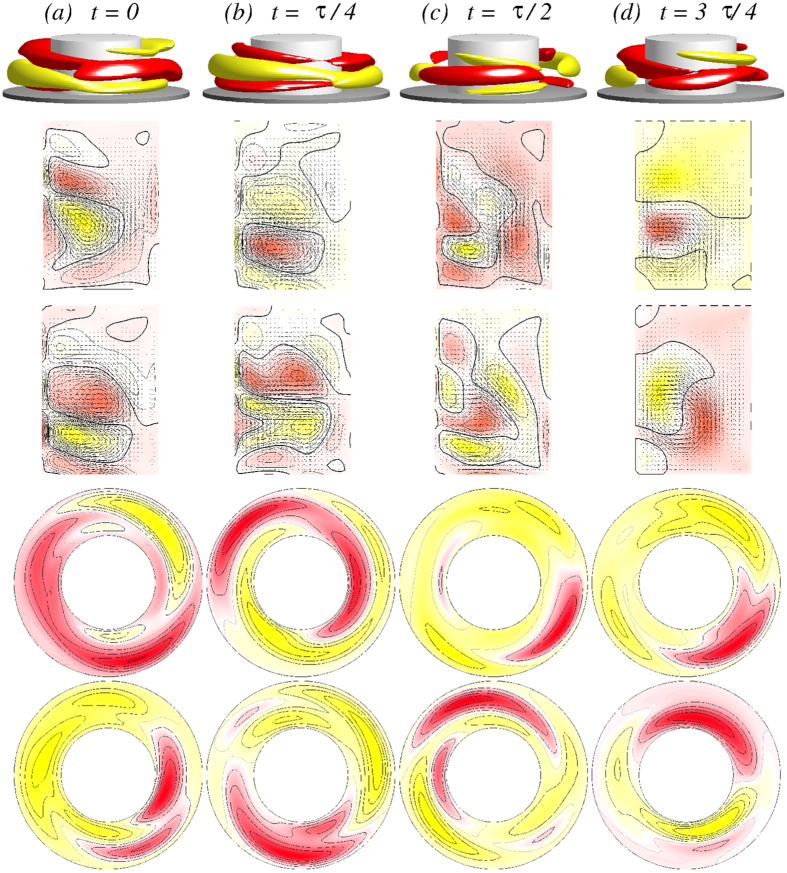
Visualization of rotating flow state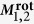 for Γ = 1.3 with the same legends as in Fig. 5. The top row shows the isosurfaces for *rv* = ±25. The period of rotation is *τ*_rot_ ≈ 0.7829. See movie file movieD1.avi and movieD2.avi in SMs. The vectorplots in (*r, z*) plane highlight very well the complexity of 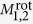, in particular the change from one-two-three-four cell states.

**Figure 14:**
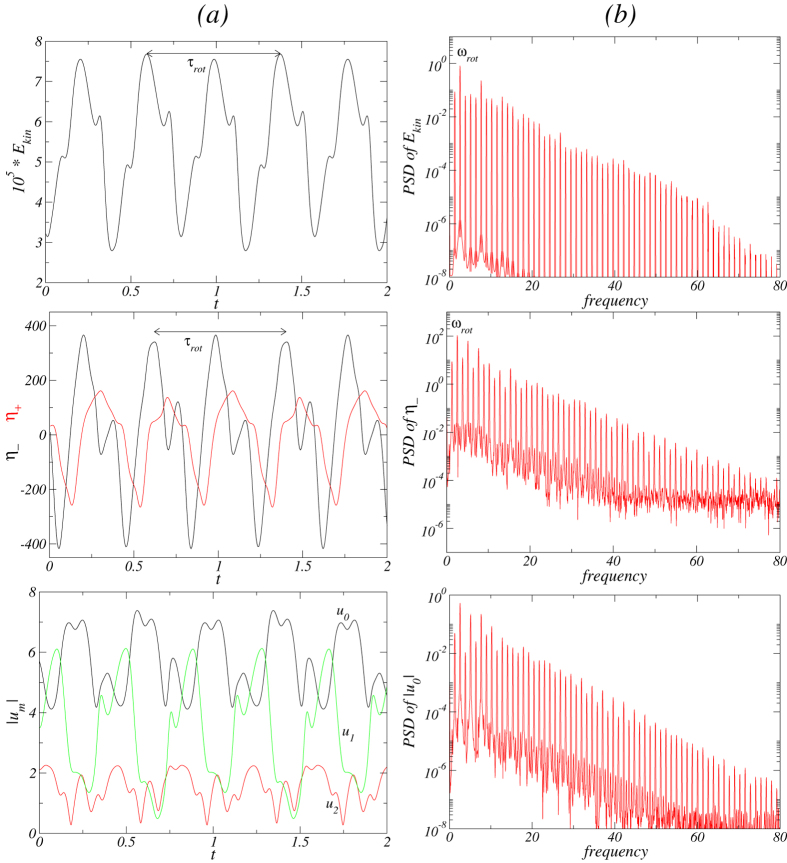
Time series and PSD of the rotating flow state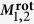. For Γ = 1.3 and *Re*_2_ = −250, (**a**) Time series of *E*_*kin*_, *η*_+_ [red (gray)], *η*_−_ (black), and the amplitudes |*u*_*m*_|, and (**b**) the corresponding PSDs. The period of rotation is *τ*_rot_ ≈ 0.7829 with the corresponding frequency *ω*_rot_ ≈ 2.554. The frequency of the underlying azimuthal oscillation is *ω*_*θ*_ (period *τ*_*θ*_), which is visible on the top of the long rotation period with about *τ*_*θ*_ ≈ *τ*_*rot*_/8 (cf. Fig. 13).

**Figure 15:**
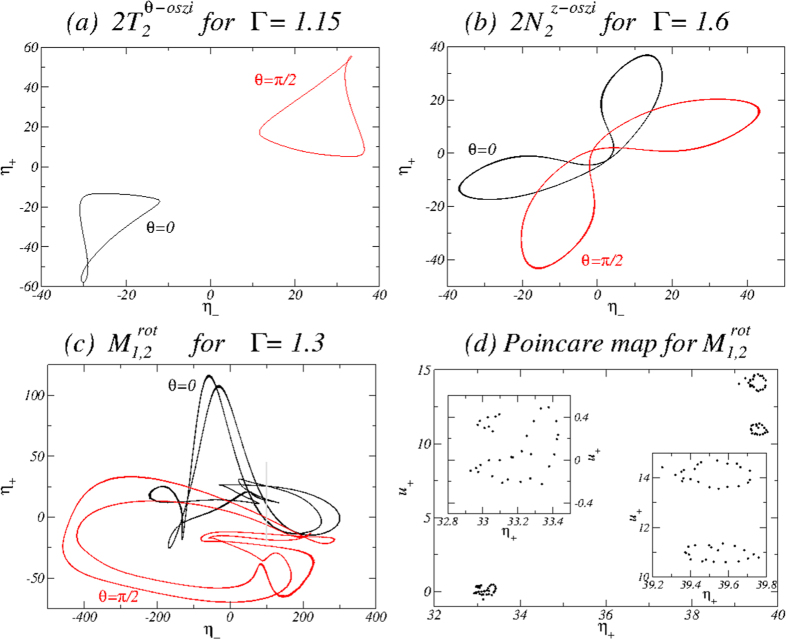
Phase portraits of the flow states for *Re*_2_ = −250. Phase portraits in the (*η*_+_, *η*_−_) plane of (**a**) 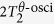 for Γ = 1.15, (**b**) 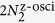 for Γ = 1.6, and (**c**) 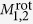 for Γ = 1.3, where *η*_±_ = *η(r*_*i*_, *θ*, ±Γ/4, *t*), and *u*_+_ = *u(d*/2, 0, Γ/4, *t*). Black [red (gray)] curves correspond to the azimuthal position *θ* = 0 [*θ* = *π*/2]. (**d**) The corresponding two-dimensional Poincaré section (*u*_+_, *η*_+_) at *η*_−_ = 200 for *θ* = 0 (gray line in (**c**)). The insets provide zoom-in views of the shown section to highlight the 2-tori characteristics of the flow state 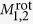.

At the bifurcation point of 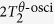, the kinetic energy 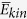 and the axisymmetric mode component *u*_0_ (cf., Fig. 2(3)) increase significantly. In the mean time the component *u*_2_ does not show any significant variation in its amplitude. Note that, up to this point, no modes other than the axisymmetric (*m* = 0) and the magnetic field induced (*m* = 2) modes have been stimulated/finite. This picture changes as Γ is increased further. In particular, for Γ ≈ 1.21, the 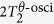 mode loses its stability when another non-axisymmetric (*m* = 1) mode emerges with a second *incommensurate* frequency *ω*_rot_ ≈ 2.48 at the onset. The flow starts to *rotate* in the azimuthal direction, so the new state 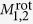 is a quasiperiodic attractor living on a 2-torus invariant manifold. 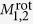 represents a relatively complex flow state in the sense that, within one period, the state can exhibit two, three and four cells, as shown in Fig. 13 [see also SMs: movieD1.avi and movieD2.avi]. Responsible for the rotation of the flow state is the *m* = 1 mode contribution, which becomes finite at the bifurcation point. This is illustrated in Fig. 14, which includes time series and the corresponding PSDs for the stimulated mode amplitudes. Regarding the time series, PSD in Fig. 14 as phase space plots (cf., Fig. 15(c,d)) the increased complexity of 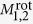 to former flow states is obious.

From the *rv* isosurfaces and the cross-section plots of *v(r, θ, z* = 1/4[1/2]Γ) in Fig. 13, the strength *u*_1_ of the *m* = 1 mode contribution can be seen. Comparing with the time series and PSDs of different characterizing quantities for the 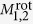 state (cf., Fig. 14), the 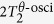 state (cf., Fig. 12) possesses a higher degree of complexity. The newly emerged frequency *ω*_rot_ is about 8 times the frequency *ω*_*θ*_, which is visible on top of the longer rotation period (cf., Fig. 14).

As Γ is increased so that the flow state changes from 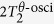 to 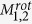, the kinetic energy 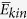 continues to increase but it is significantly smaller than that for the 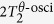 state (cf., Fig. 2(2)). In the meantime, the emergence (from zero) and enhancement of the *m* = 1 mode (*u*_1_) is compensated by a decrease in the strength of the axisymmetric *m* = 0 mode (*u*_0_), with that of the *m* = 2 mode (*u*_2_) decreasing only slightly. As for *Re*_2_ = 0, there exists a regime, 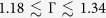, in which the non-connected solutions for one- and two-cell flow states bistable coexist.

Figure 15 shows the phase portraits of the different time-dependent flow states 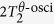, 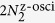 and 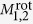 on the (*η*_+_, *η*_−_) plane for *Re*_2_ = −250 and values of Γ as indicated. A Poincaré section (*u*_+_, *η*_+_) is also used to better visualize the quasiperiodic nature of the flow state 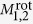. We see that only the oscillating states 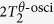 and 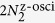 possess limit cycle characteristics while the Poincaré section (*u*_+_, *η*_+_) highlights the two-tori characteristics of the 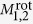 state with two incommensurate frequencies, i.e., the azimuthal oscillation frequency *ω*_*θ*_ and the rotation frequency *ω*_rot_. Note that the curves in (*u*_+_, *η*_+_) (Fig. 15(*d*)) do not fully close on themselves due to the long returning times to the section. The insets present a zoom-in view of the parameter region. The phase portraits illustrate the shift-reflect symmetry of the 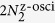 state. From the phase portrait for 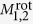, an increased level of complexity due to the additional rotation of the flow state can be seen.

#### The case of *Re*_2_ = −500

We now study the case of strongly counter rotating cylinders: *Re*_2_ = −500. The bifurcation diagram with Γ is shown in Fig. 2(4). For Γ = 1.6 we find the flow state 2*N*_2_ (Fig. 2(1)) Increasing Γ, the state loses its stability at Γ ≈ 1.62 through a symmetry breaking Hopf bifurcation and the resulting state is again the unsteady, axially oscillating flow state 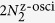 (cf., Figs 5 and 6), which remains stable until Γ ≈ 1.75.

We find that the oscillation amplitude is much smaller than that for the 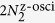 state at *Re*_2_ = −250 (cf. Fig. 5). As Γ is decreased, the state remains stable until for Γ ≈ 1.18 when it loses stability, at which the one-cell flow state 1*A*_2_ (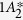) emerges, similar to the cases of *Re*_2_ = 0 and *Re*_2_ = −250. In the opposite direction, i.e., starting from the steady flow state 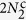 for Γ = 0.5 and increasing Γ, the state loses its stability at Γ ≈ 0.59 through the same symmetry breaking pitchfork bifurcation as for the cases of *Re*_2_ = 0 and *Re*_2_ = −250. Due to the stronger counter rotation, two additional vortex cells start to develop near the outer cylinders for the flow state 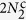. The symmetry related one-cell flow state 1*A*_2_ or 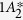 appears as the size of one of the vortex cells increases or shrinks, respectively. Upon further increase in Γ, the flow state 1*A*_2_ remains stable with slight but continuous change in the position of the vortex cell. The larger vortex cell moves towards the inner cylinder, while the second vortex cell grows and moves radially outward towards the outer cylinder. In principle this is the same evolution as for the case of *Re*_2_ = −250, with the only difference being that, due to the stronger counter rotation (*Re*_2_ = −500), the vortex cells and in particular their centers are slightly shifted and located closer towards the inner cylinder.

For Γ ≈ 1.08, the flow becomes time dependent, and the azimuthally oscillating twin-cell flow state 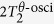 emerges. The dynamics is almost the same as seen in Figs 11 and 12 for slightly smaller Γ = 1.15 and *Re*_2_ = −250. With a further increase in Γ, this state loses its stability at Γ ≈ 1.35 and becomes a transient to the flow state 2*N*_2_. For *Re*_2_ = −500, there then exists again a regime, 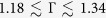, in which the one-cell and two-cell flow states bistable coexist. Differing from the case of *Re*_1_ = −250, there is absence of more complex (e.g., quasiperiodic solution) flow state for *Re*_2_ = −500. The phase portraits for the azimuthally and axially oscillating flow states 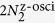 and 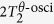 is similar to these solutions at *Re*_2_ = −250.

A focus of our present study is axially or azimuthally oscillating flow states. It should be noted, however, that flow states of *combined* axial and azimuthal oscillations can occur in the parameter regime of larger aspect ratio and very large values of the Reynolds number. A more detailed discussion about the behaviors of the angular momentum and torque can be found in Supplementary Materials.

## Discussion and Summary of Main Findings

As a fundamental paradigm of fluid dynamics, the TCS has been extensively investigated computationally and experimentally. In spite of the long history of the TCS and the vast literature on the subject, the dynamics of TCS with a complex fluid subject to a symmetry breaking magnetic field have begun to be investigated relatively recently. In fact, a gap existed in our knowledge about the nonlinear dynamics of such systems with a small aspect ratio. The present work is aimed to fill this gap.

Through systematic and extensive simulations of the ferrohydrodynamical equations, a generalization of the classic Navier-Stokes equation into ferrofluidic systems subject to a magnetic field, we unveil the emergence and evolution of distinct dynamical flow states. As the Reynolds number of the outer cylinder and/or the aspect ratio is changed, symmetry-breaking pitchfork and Hopf bifurcations can occur, leading to transitions among various flow states, e.g., the two-cell and one-cell states. The presence of the transverse magnetic field stipulates that all flow states must inherently be three-dimensional^5^,^7^,^43^.

The detailed emergence, dynamical evolution, and transitions among the various flow states can be summarized, as follows. We first identify a fundamental building block that plays a dominant role in the formation of various flow structures: the order-two azimuthal *m* = 2 mode. For small aspect ratio (e.g., Γ ≈ 0.5), the two-cell state 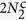 dominates which, due to its two-fold flow symmetry, differs little from the one in the classical TCS^34^. Depending on the rotational speed and the direction of the outer cylinder the vortex cells within the 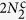 state can move closer towards the inner cylinder. The flow is steady and exhibits a more complex set of symmetries associated with the magnetic field, namely, a combination of the two-fold symmetry and the reflection symmetry about the mid-plane under reversal of the field direction. As Γ is increased, this flow state undergoes a symmetry breaking bifurcation at which one vortex cell starts to grow while the other begins to shrink, eventually generating two symmetry related one-cell flow states: 1*A*_2_ and 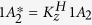. When the outer cylinder is at rest (i.e., *Re*_2_ = 0), the state 1*A*_2_ loses stability and eventually becomes a transient to the steady, axially symmetric two-cell flow state 2*N*_2_. For counter-rotating cylinders (i.e., *Re*_2_ < 0), we find a transition to the same flow state 2*N*_2_ at a larger value of Γ. However, prior to this transition a distinct bifurcation sequence leading to *new unsteady* flow states occurs. In fact, as Γ is increased, the one-cell flow state 1*A*_2_ becomes modulated in that the smaller vortex cell grows along the inner cylinder while the other vortex cell is pulled outward. Eventually the steady flow state 1*A*_2_ undergoes a Hopf bifurcation to a periodic, *azimuthally oscillating* flow state 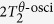 in the twin-cell configuration (side-by-side arrangement) where both vortex cells touch the top and bottom lids, which topologically corresponds to a limit cycle. During the dynamical evolution, there are two symmetry related flow states: 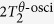 and 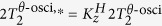. Increasing Γ further, we find two possible bifurcation scenarios. First, 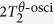 loses its stability and becomes a transient to the steady flow state 2*N*_2_. Second, 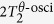 becomes unstable, leading to an unsteady quasiperiodic flow state 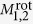. The quasiperiodic state has finite contribution from the *m* = 1 mode and rotates in the azimuthal direction. As a result, one of the two frequencies, *ω*_*θ*_, corresponds to the frequency of the underlying 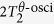 mode from which it bifurcates, and the second frequency *ω*_rot_ comes from the rotation of the *m* = 1 mode, a flow state with a helical shape (cf., Fig. 13). For larger values of Γ, the unsteady flow state 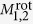 eventually loses its stability and becomes transient towards the steady state 2*N*_2_.

A similar scenario occurs when the aspect ratio is varied in the opposite direction, i.e., from large to small values. At a certain point the steady two-cell flow state 2*N*_2_ loses its stability and is replaced by one of the symmetry related steady one-cell flow states, 1*A*_2_ or 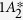. Depending on the parameters there is a relatively large regime in which the both not-connected solutions, two-cell and one-cell flow states bistable coexist. It is worth mentioning that our computations never reveal any signature of the transition from the steady two-cell flow state 2*N*_2_ to any of the unsteady one-cell flow states (i.e., 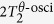 or 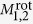). The reduction in the vortex cells (from two to one) appears to happen only between the steady flow states.

In addition to these unsteady flow states, we detect another unsteady flow state, the axially oscillating flow state 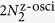 that is known for the classical TCS^34^. The state 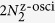 emerges at a large value of Γ or through variation of the rotation speed of the outer cylinder through a symmetry breaking Hopf bifurcation out of the flow state 2*N*_2_. Similar to 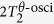, the flow state 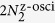 is a limit cycle solution which is half-period flip invariant under the symmetry operation *S*^*H*^.

**Figure 16:**
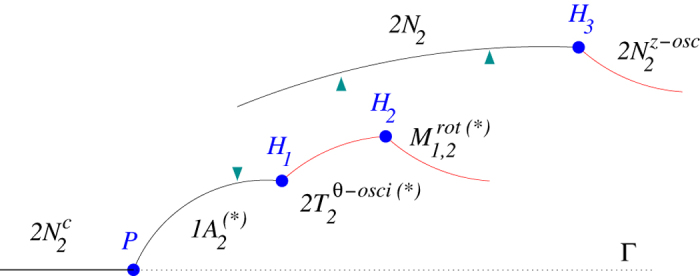
Summary: schematic bifurcations for ferrofluidic TCS with a small aspect ratio. For illustrative purpose, the aspect ratio Γ is taken as the bifurcation parameter, where *P* denotes the pitchfork bifurcation of 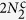 into two symmetry related flow states 1*A*_2_ and 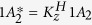. The Hopf bifurcation *H*_1_ generates the limit cycle solution 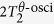 (or 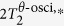). At the Hopf bifurcation *H*_2_, a quasiperiodic symmetric flow state 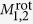 (or 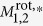) is born out of the limit cycle. Depending on other system parameters the flow becomes transient towards the 2*N*_2_ state from either 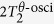 or 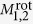. The state 2*N*_2_ undergoes another Hopf bifurcation *H*_3_, generating a distinct limit cycle solution, 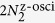. Black [Red (gray)] colored lines indicate steady [unsteady] flow states.

To summarize the complicated bifurcation/transition scenarios in the ferrofluidic TCS with a small aspect ratio in a transparent way, we produce a schematic bifurcation diagram with the aspect ratio Γ being the bifurcation parameter, as shown in Fig. 16. The symmetry breaking associated with each bifurcation point can be described succinctly, as follows. At the pitchfork bifurcation point *P*, the two-cell flow state 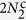, which is invariant under the symmetries 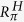 and 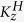, loses stability, giving rise to the one-cell flow state 1*A*_2_. In fact, breaking the 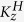 symmetry results in two symmetry related flow states 1*A*_2_ and 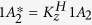. The state 1*A*_2_ loses stability through the Hopf bifurcation *H*_1_ at which a limit cycle state 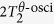 (or 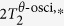) is born. Finally a second frequency appears through another Hopf bifurcation *H*_2_, leading to a two-frequency quasiperiodic solution 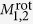 (or 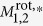).

## Methods

### System setting and the Navier-Stokes equation

We consider a standard TCS consisting of two concentric, independently rotating cylinders. Within the gap between the two cylinders there is an incompressible, isothermal, homogeneous, mono-dispersed ferrofluid of kinematic viscosity *ν* and density *ρ*. The inner and outer cylinders have radius *R*_1_ and *R*_2_, and they rotate with the angular velocity *ω*_1_ and *ω*_2_, respectively. The boundary conditions at the cylinder surfaces are of the non-slip type, and the end walls enclosing the annulus are stationary. The system can be characterized in the cylindrical coordinate system (*r, θ, z*) by the velocity field **u** = (*u, v, w*) and the corresponding vorticity field ∇ × **u** = (*ξ, η, ζ*). We fix the radius ratio of the cylinders: *R*_1_/*R*_2_ = 0.5, and vary the height-to-gap aspect ratio of the annulus in the range 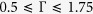. A homogeneous magnetic field **H** = *H*_*x*_**e**_*x*_ is applied in the transverse (*x* = *r* cos *θ*) direction, with *H*_*x*_ being the field strength. The length and time scales of the system are set by the gap width *d* = *R*_2_ − *R*_1_ and the diffusion time *d*^2^/*ν*, respectively. The pressure in the fluid is normalized by *ρν*^2^/*d*^2^, and the magnetic field **H** and the magnetization **M** can be conveniently normalized by the quantity 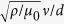, where *μ*_0_ is the permeability of free space. These considerations lead to the following set of non-dimensionalized hydrodynamical equations^7^,^44^:


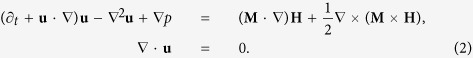


The boundary conditions are set as follows. The velocities at the stationary boundaries (i.e., lids) are zero. On the cylindrical surfaces, the velocity fields are given by **u**(*r*_1_, *θ, z*) = (0, *Re*_1_, 0) and **u**(*r*_2_, *θ, z*) = (0, *Re*_2_, 0), where the inner and outer Reynolds numbers are *Re*_1_ = *ω*_1_*r*_1_*d/ν* (fixed at 250 in the present study) and *Re*_2_ = *ω*_2_*r*_2_*d/ν*, respectively, where *r*_1_ = *R*_1_/(*R*_2_ − *R*_1_) and *r*_2_ = *R*_2_/(*R*_2_ − *R*_1_) are the non-dimensionalized inner and outer cylinder radii, respectively. Note that the idealized boundary conditions are discontinuous at the junctions where the stationary end walls meet the rotating cylinders. In experiments there are small but finite gaps at these junctions where the azimuthal velocity adjusts to zero. To treat the boundary conditions properly, we implement the following regularization procedure for the boundary conditions:


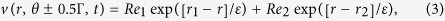


where *ε* is a small parameter characterizing the physical gaps. In the simulations, we set *ε* = 6 × 10^−3^. The range of variation in *Re*_2_ is −2000 ≤ *Re*_2_ ≤ 500.

### Ferrohydrodynamical equation

Equation (2) is to be solved together with an equation that describes the magnetization of the ferrofluid. Using the equilibrium magnetization of an unperturbed state in which the homogeneously magnetized ferrofluid is at rest and the mean magnetic moment is orientated in the direction of the magnetic field, we have **M**^eq^ = *χ***H**. The magnetic susceptibility *χ* of the ferrofluid can be approximated by the Langevin’s formula^45^, where we set the initial value of *χ* to be 0.9 and use a linear magnetization law. The ferrofluid studied corresponds to APG933^46^. We consider the near equilibrium approximations of Niklas^47^,^48^ with a small value of ||**M** − **M**^eq^|| and small magnetic relaxation time *τ*: |∇ × **u**|*τ* ≪ 1. Using these approximations, one can obtain^7^ the following magnetization equation:


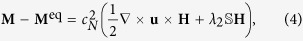


where


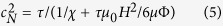


is the Niklas coefficient^47^, *μ* is the dynamic viscosity, Φ is the volume fraction of the magnetic material, 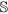 is the symmetric component of the velocity gradient tensor^7^,^44^, and *λ*_2_ is the material-dependent transport coefficient^44^ that can be conveniently chosen to be^3^,^44^,^49^ *λ*_2_ = 4/5. Using Eq. (4), we eliminate the magnetization from Eq. (2) to arrive at the following ferrohydrodynamical equations 7,44:


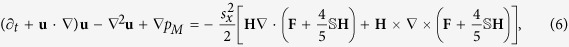


where **F** = (∇ × **u**/2) × **H**, *p*_*M*_ is the dynamic pressure incorporating all magnetic terms that can be expressed as gradients, and *s*_*x*_ is the Niklas parameter [Eq. (8)]. To the leading order, the internal magnetic field in the ferrofluid can be approximated by the externally imposed field^36^, which is reasonable for obtaining the dynamical solutions of the magnetically driven fluid motion. Equation (6) can then be simplified as


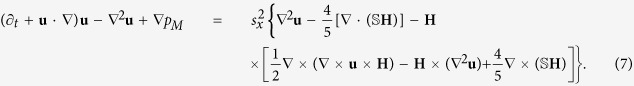


This way, the effect of the magnetic field and the magnetic properties of the ferrofluid on the velocity field can be characterized by a single parameter, the magnetic field or the Niklas parameter^47^:


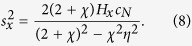


### Numerical method

The ferrohydrodynamical equations of motion Eq. (6) can be solved^3^,^7^,^36^ by combining a standard, second-order finite-difference scheme in (*r, z*) with a Fourier spectral decomposition in *θ* and (explicit) time splitting. The variables can be expressed as


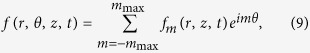


where *f* denotes one of the variables {*u, v, w, p*}. For the parameter regimes considered, the choice 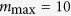 provides adequate accuracy. We use a uniform grid with spacing *δr* = *δz* = 0.02 and time steps *δt* < 1/3800.

### Symmetries

In a classical TCS or a ferrofluidic TCS without any external magnetic field where the fluid is confined by end walls, the system is invariant with respect to arbitrary rotations about the axis and the reflections about axial mid-height. For a ferrofluid under a transverse magnetic field, these symmetries are broken and the flow is inherently three-dimensional for any non-zero values of the parameters *Re*_1_, *Re*_2_ and *s*_*x*_, due to the rotation of the cylinders^5^,^7^,^36^,^43^. With at least one cylinder rotating, the inclusion of the magnetic terms in the ferrohydrodynamic equation results in a downward directed force on the side where the field enters the system (*θ* = 0), and an upward directed force on the opposite side (*θ* = *π*) where the field exits the annulus. The resulting flow states can possess more complicated symmetries, such as the reflection 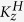 about the annulus mid-height plane along with an inversion of the magnetic field direction. There can also be a rotational invariance 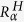 for discrete angle *α* = *π* in combination with the reversal of the magnetic field, where the angle *π* specifies the direction of the magnetic field when entering the annulus. Thus the symmetries associated with the velocity field are


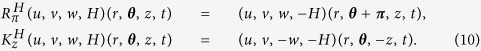


For a periodic solution (with period *τ*), the flow field is also invariant under the discrete time translation


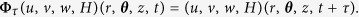


Further details of the magnetic field induced two-fold symmetry can be found in ref. 7.

## Additional Information

**How to cite this article**: Altmeyer, S. *et al*. Dynamics of ferrofluidic flow in the Taylor-Couette system with a small aspect ratio. *Sci. Rep.* **7**, 40012; doi: 10.1038/srep40012 (2017).

**Publisher's note:** Springer Nature remains neutral with regard to jurisdictional claims in published maps and institutional affiliations.

## Supplementary Material

Supplementary Movie A1

Supplementary Movie A2

Supplementary Movie A3

Supplementary Movie A4

Supplementary Movie B1

Supplementary Movie B2

Supplementary Movie B3

Supplementary Movie B4

Supplementary Movie C1

Supplementary Movie C2

Supplementary Movie C3

Supplementary Movie D1

Supplementary Movie D2

Supplementary Movie E1

Supplementary Movie E2

Supplementary Movie E3

Supplementary Material

## Figures and Tables

**Table 1:** Flow state nomenclature and abbreviations.

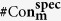		
Indicator	Description	Elements
#	number of vortex cells	1, 2
**Con**	configuration	**N** (normal),
		**A** (anomalous),
		**T** (twin-cell)
		**M** (modulated rotating wave),
**spec**	specification	**z-osci** (axially oscillating),
		*θ***-osci** (azimuthally oscillating),
		**rot** (rotating)
		**c** (compressed)
		* (symmetry related)
**m**	stimulated modes	1, 2
