# Supplementary material for: Dynamics of ferrofluidic flow in the Taylor-Couette system with a small aspect ratio
Source: Sci Rep. 2017 Jan 6;7:40012. doi: 10.1038/srep40012 (PMC5216369; doi:10.1038/srep40012)
Supplement: Supplementary Material [file srep40012-s17.pdf]

Supplementary Material to
"Dynamics of ferrofluidic flow in the Taylor-Couette system with a small aspect ratio"

Sebastian Altmeyer,[1,*] Younghae Do,[2,†] and Ying-Cheng Lai[3]

[1] *Institute of Science and Technology Austria (IST Austria), 3400 Klosterneuburg, Austria*
[2] *Department of Mathematics, KNU-Center for Nonlinear Dynamics, Kyungpook National University, Daegu, 41566, Republic of Korea*
[3] *School of Electrical, Computer and Energy Engineering, Arizona State University, Tempe, Arizona, 85287, USA*

(Dated: October 29, 2016)

Herewith we provide additional material, figures and movies as well as more detailed discussions for the further interested reader.

Additional figures in SM

Flow structures Figure 1 presents a slightly elongated two-cell flow state $2_L N_2$. This flow state is found to exist only at strong counterrotation ($Re \lesssim -1680$) and differs from $2N_2$ in that way that the vortex centers are pushed towards the inner cylinder and the cells become elongated in the axial direction.

Figure 2 shows visualization of the flow pattern for $\Gamma = 1.0$, $Re_2 = -250$ indicates a modulation in the flow structure of $1A_2$. The one-cell flow state remains but a minor, second vortex cell starts to grow in the axial direction near the inner cylinder as the large vortex cell (top in Fig. 2) is pulled outwards due to the counter rotation.

A typical flow state $2N_2$ is presented in Fig. 3 for parameters at $\Gamma = 1.6$ and $Re_2 = -250$. Compared to other presented $2N_2$ states for $Re = 0$ (see Fig. 3 in main paper) or $Re = -250$ (see Fig. 2) the vortex centers are shifted closer toward the inner cylinder due to the strong counter rotation.

The figures 4 and 5 present either snapshots and time series of the $2N_2^{z\text{-osci}}$ state for parameters at $\Gamma = 1.7$ and $Re_2 = -500$ (see Figs. 5 and 6 in main paper).

Figure 6 presents the flow state $1A_2$ at $\Gamma = 1.0$ and $Re_2 = -250$. Increasing Γ this flow remains first stable with slight but continuous change in the position of the vortex cell. The larger vortex cell moves towards the inner cylinder, while the second vortex cell grows and moves radially outward towards the outer cylinder. In principle this is the same evolution as for the case of $Re_2 = -250$, with the only difference being that, due to the stronger counter rotation ($Re_2 = -500$), the vortex cells and in particular their centers are slightly shifted and located closer towards the inner cylinder.

The Figs. 7 and 8 present snapshots, time series and PSD for the azimuthally oscillating twin-cell flow state $2T_2^{\theta\text{-osci}}$ at $\Gamma = 1.2$, $Re_2 = -500$.

Behaviors of the angular momentum and torque To further characterize the flow states, we examine the behaviors of the angular momentum and torque. Figure 10 shows the mean (axially and azimuthally averaged) angular momentum $L(r) = r\langle v(r)\rangle_{\theta,z}/Re_1$, normalized by the inner Reynolds number Re_1, versus the radius r for different values of the aspect ratio Γ. For unsteady flow states, the time-averaged values over one period are shown, where the gray thin solid line indicates the behavior for the unstable equilibrium circular Couette flow (CCF) for comparison. For all the flow states, the angular momentum is transported outwards from the inner cylinder, which is typical for the TCS.

When the outer cylinder is at rest, the $L(r)$ curves have a large slope near the inner cylinder wall. For the $2N_2^c$ state the curve is convex. The largest slope of $L(r)$ at the inner boundary corresponds to the smallest value of Γ. After the bifurcation leading to the emergence of the one-cell flow state $1A_2$, the $L(r)$ curves start to form a plateau region about the center of the bulk, which becomes more pronounced as Γ is increased, as shown in Fig. $10(a)$. The fact that for the flow state $2N_2$ the curve $L(r)$ has a local maximum in the outer bulk region at $r \approx 0.72d$ indicates strongly oscillatory dynamics in the outer region. For $Re_2 < 0$ all $L(r)$ curves have a similar shape with increased slope near the boundaries and reduced slope in the interior. Due to the stronger torque the steepest part of $L(r)$ now occurs at the outer boundary. Increasing Γ the slopes of $L(r)$ near the inner and outer boundaries decrease. For $Re = -250$, changes in the shape of the $L(r)$ curves are relatively moderate, where the largest change occurs at $r \approx 0.35d$ and $r \approx 0.85d$ while close to the central region ($r \approx 0.6d$), there is little change in $L(r)$ (pinned). For the two-cell flow states $2N_2$ and $2N_2^{z\text{-osci}}$, the central region is flattened. For $Re = -500$ the variations of the flow states in the outer half of the bulk are the strongest. Increasing Γ results in a decrease in the slope of $L(r)$, minimizing the size

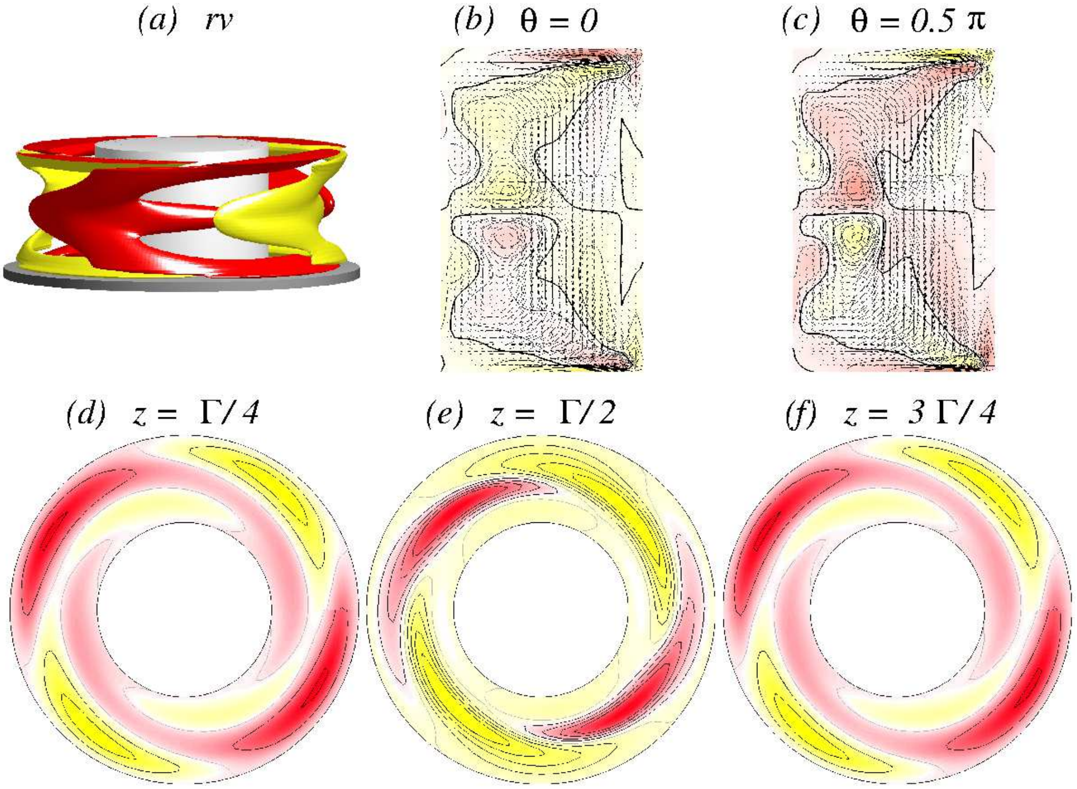

FIG. 1: **Visualization of the flow state $2_L N_2$.** Flow visualization of $2_L N_2$ for $\Gamma = 1.6$, $Re_2 = -2000$: (a) isosurface of rv (isolevel shown at $rv = \pm 7$) and the corresponding vector plot $[u(r,z), w(r,z)]$ of the radial and axial velocity components (including the azimuthal vorticity $\eta(r,\theta)$) for (b) $\theta = 0$ and (c) $\theta = \pi/2$. (d-f) The azimuthal velocity $v(r,\theta)$ in three different planes: $z = \Gamma/4$, $z = \Gamma/2$, and $z = 3\Gamma/4$, respectively. The same legends are used for visualizing all the time independent flows in the paper.

of the interior plateau region. Similar to the case of $Re = -250$, the central parts of the curve $L(r)$ for the two-cell flow states lie below the ones for the one-cell states.

After the averaged quantities we will now look at the variations within the time-dependent solutions. Therefore Fig. 11 shows the variation in the angular momentum $L(r)$ over one period for different unsteady state flows. We see that variations of $L(r)$ for the axially oscillating flow state $2N_2^{z\text{-osci}}$ are quite small. Moderate changes over one period are visible only in the inner half of the bulk for $Re_2 = -250$, as shown in Fig. $11(a)$. For $Re_2 = -500$ the variations are insignificant, as shown in Fig. $11(b)$. However, for the azimuthally oscillating flow states $2T_2^{\theta\text{-osci}}$, the variations in $L(r)$ over one period are moderate (larger than those for axial oscillations) with stronger (weaker) amplitude for $Re_2 = -250$ ($Re_2 = -500$). Over one period the slope of $L(r)$ in the interior region changes significantly. Modulation also takes place near the center of the bulk, as shown in Fig. $11(c)$. For the rotating flow state $M_{1,2}^{\text{rot}}$, the main changes in $L(r)$ occurs in the outer bulk region, as shown in Fig. $11(e)$, accounting for the dynamics associated with the $m = 1$ mode.

Last we will investigate on the behaviors of the dimensionless torque $G = \nu J^{\omega}$ with Re_2 and Γ (Fig. 12). The torque is calculated based on the fact that, for a flow between infinite cylinders the transverse current of the azimuthal motion, i.e., $J^{\omega} = r^3[\langle u\omega\rangle_{A,t} - \nu\langle\partial_r\omega\rangle_{A,t}]$ [1], where $\langle ...\rangle_A \equiv \int \frac{rd\theta dz}{2\pi rl}$, is a conserved quantity [1]. Thus, the dimensionless torque is the same at the inner and outer cylinders. For the two-cell and four-cell flow states for $\Gamma = 1.6$ (Fig. 11), the torque G is minimal for $Re_2 = 0$ and increases monotonically as the value of Re_2 is increased in either direction. Note that we do not distinguish the various flow states in detail but only focus on the one-cell and two-cell flow states. For $Re = 0$ ($Re = -500$), the torque G monotonically increases (decreases) as Γ is increased, regardless of the nature of the flow state (e.g., one-cell, two-cell, steady, or unsteady). For $Re = -250$ the torque for the one-cell flow states initially decreases with Γ, reaches a minimum and increases afterwards. However for the two-cell flow states, G increases monotonically with Γ.

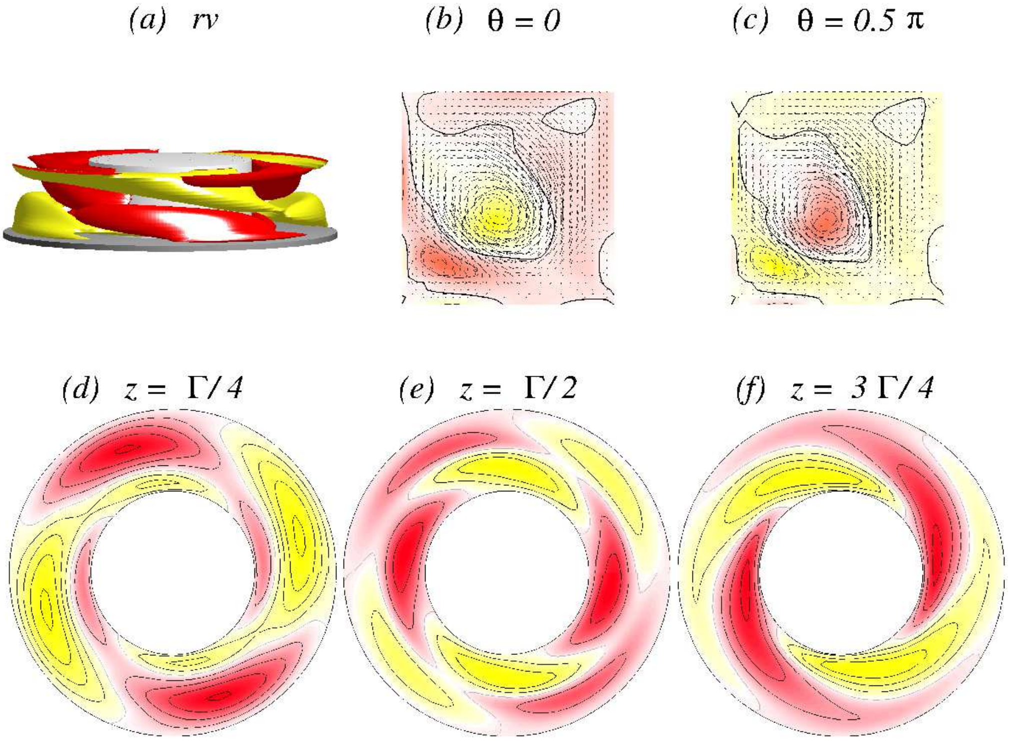

FIG. 2: **Visualization of flow state** $1A_2$ for $\Gamma = 1.0$ and $Re_2 = -250$: The isosurface for $rv = \pm 7$ is shown in (a). The legends are the same as in Fig. 1.

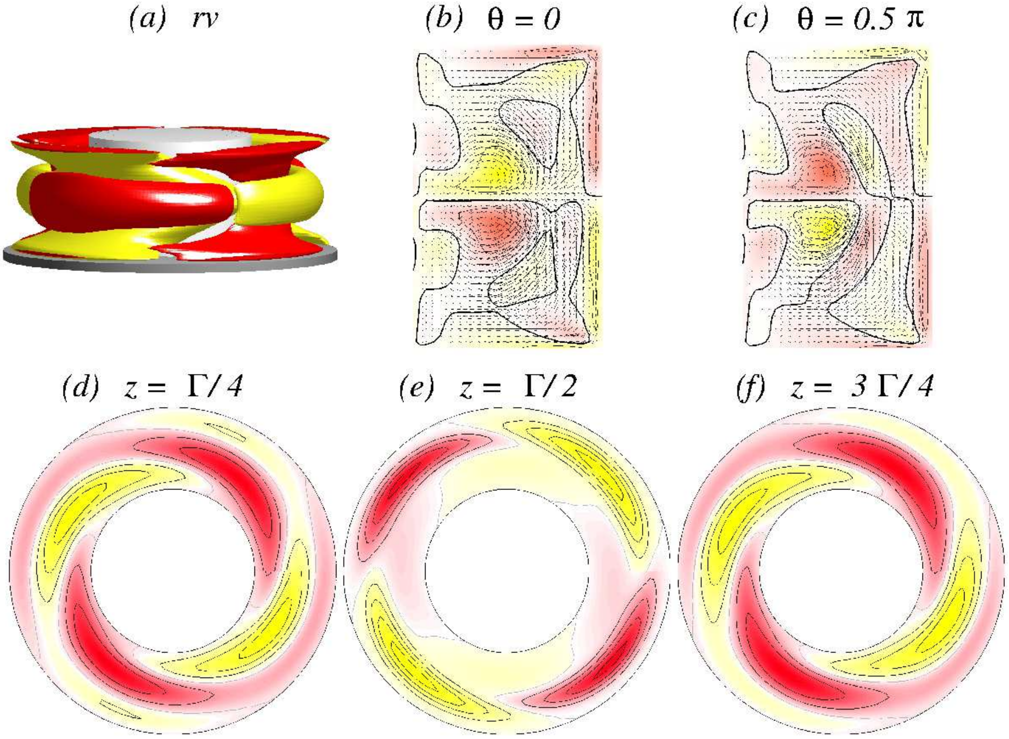

FIG. 3: **Visualization of flow state** $2N_2$ for $\Gamma = 1.6$ and $Re_2 = -500$. The isosurface for $rv = \pm 7$ is shown in (a). The legends are the same as in Fig. 1. Compared with the $2N_2$ state for $Re = 0$ [Fig. 3 in main paper], the vortex centers are shifted closer toward the inner cylinder due to the strong counter rotation.

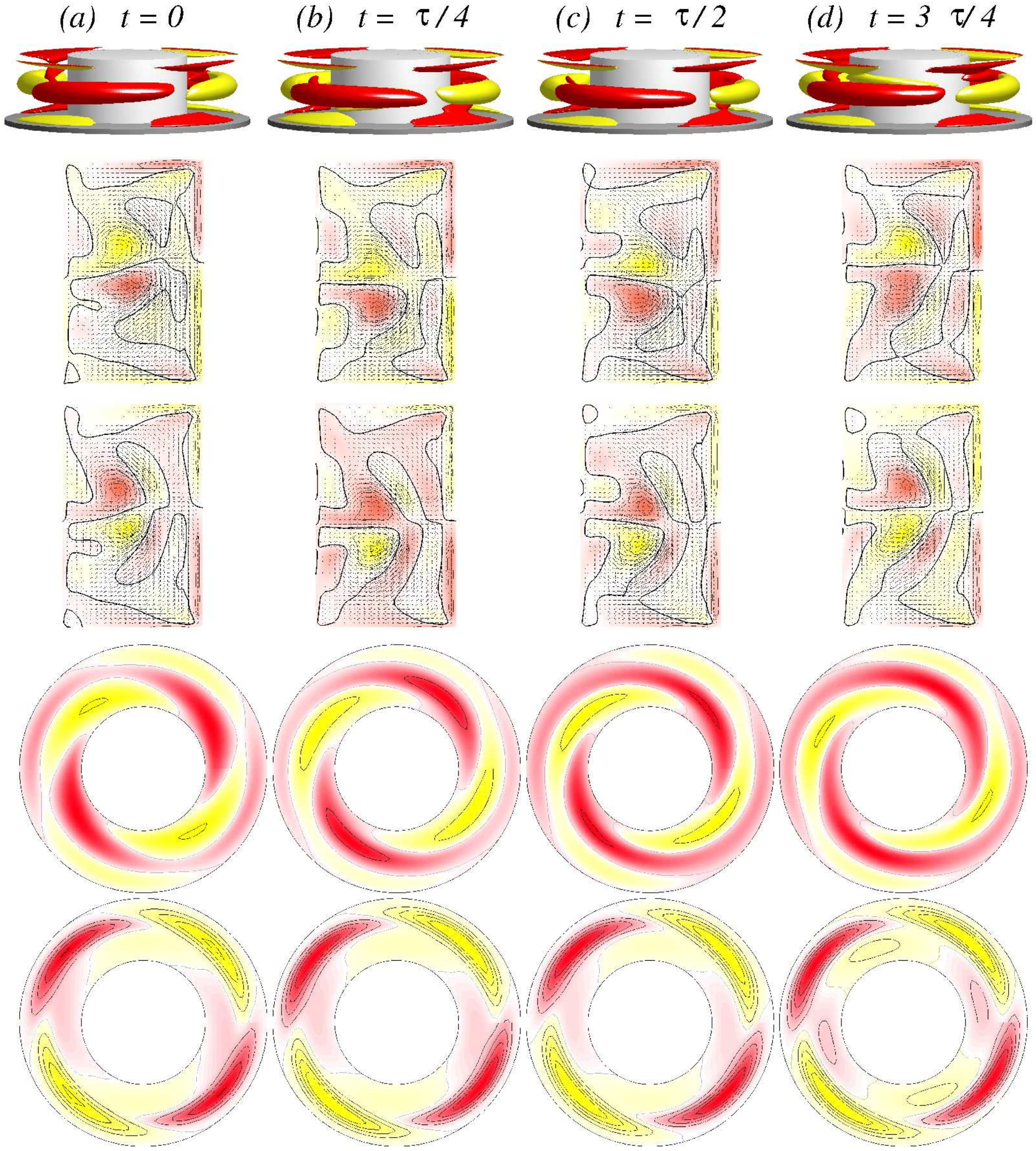

FIG. 4: **Visualization of the axially oscillating two-cell flow state** $2N_2^{\textbf{z-osci}}$. The first row shows, for $\Gamma = 1.7$ and $Re_2 = -500$, the isosurfaces of rv (isolevel shown at $rv = \pm 15$) over one axially oscillating period ($\tau_z \approx 0.157$, and corresponding frequency $\omega_\theta \approx 12.682$) at instants of time t as indicated. The second and third rows show the corresponding vector plots $[u(r,z), w(r,z)]$ of the radial and axial velocity components in the planes defined by $\theta = 0$ and $\theta = \pi/2$, respectively, where the color-coded azimuthal vorticity field η is also shown. The fourth and fifth rows represent the azimuthal velocity $v(r,\theta)$ in the axial planes $z = \Gamma/4$ and $z = \Gamma/2$, respectively. Red (dark gray) and yellow (light gray) colors correspond to positive and negative values, respectively, with zero specified as white. See also movie file movieA1.avi in Supplementary Materials (SMs). The same legends for flow visualization are used for all subsequent unsteady flows. See movie files movieE1.avi, movieE2.avi and movieE3.avi in Supplementary Materials. Comparing with the $2N_2^{\text{z-osci}}$ state [Fig. 5 in main paper], the oscillation amplitude is smaller.

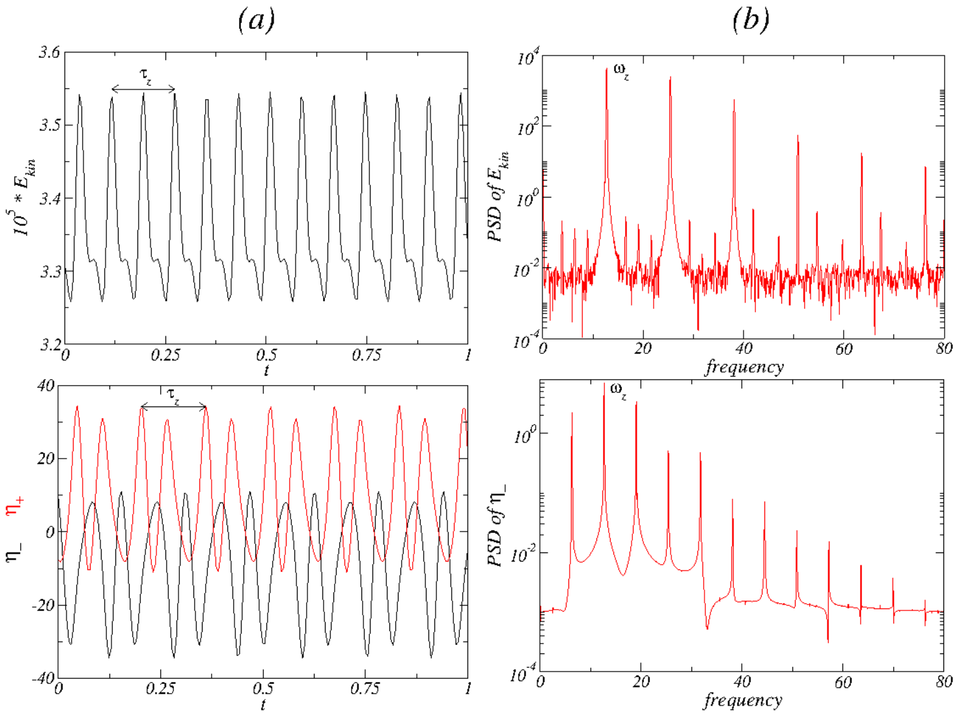

FIG. 5: **Time series and PSDs for axially oscillating two-cell flow** $2N_2^{\text{z-osci}}$**.** For $\Gamma = 1.7$ and $Re_2 = -500$, (a) Time series of E_{kin}, η_+ [red (gray)], and η_- (black). (b) The corresponding PSDs for the $2N_2^{\text{z-osci}}$ state. The period of azimuthal oscillation is $\tau_\theta \approx 0.157$ with the corresponding frequency $\omega_\theta \approx 12.682$. The peak in the PSD of η_- at $\omega_\theta/2$ indicates the half-period flip symmetry of the $2N_2^{\text{z-osci}}$ state [see Fig. 13 in main paper].

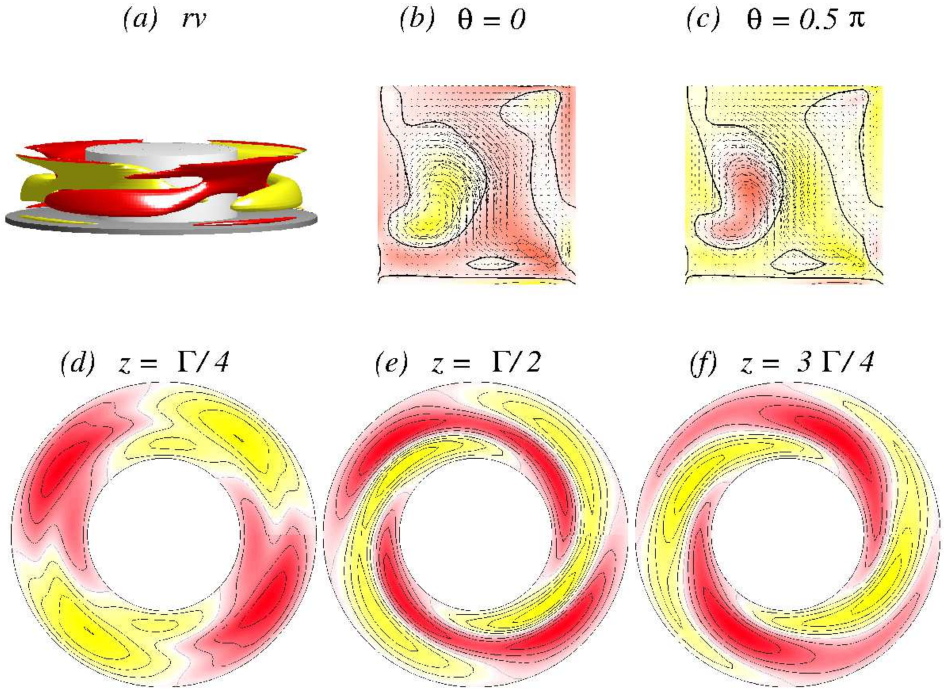

FIG. 6: **Visualization of flow state** $1A_2$ for $\Gamma = 1.0$ and $Re_2 = -500$, where panel (a) shows the isosurface for $rv = \pm 12$. Legends are the same as in Fig. 1. The evolution from a one-cell towards a twin-cell flow state can be seen as the 2nd vortex expands in the axial direction.

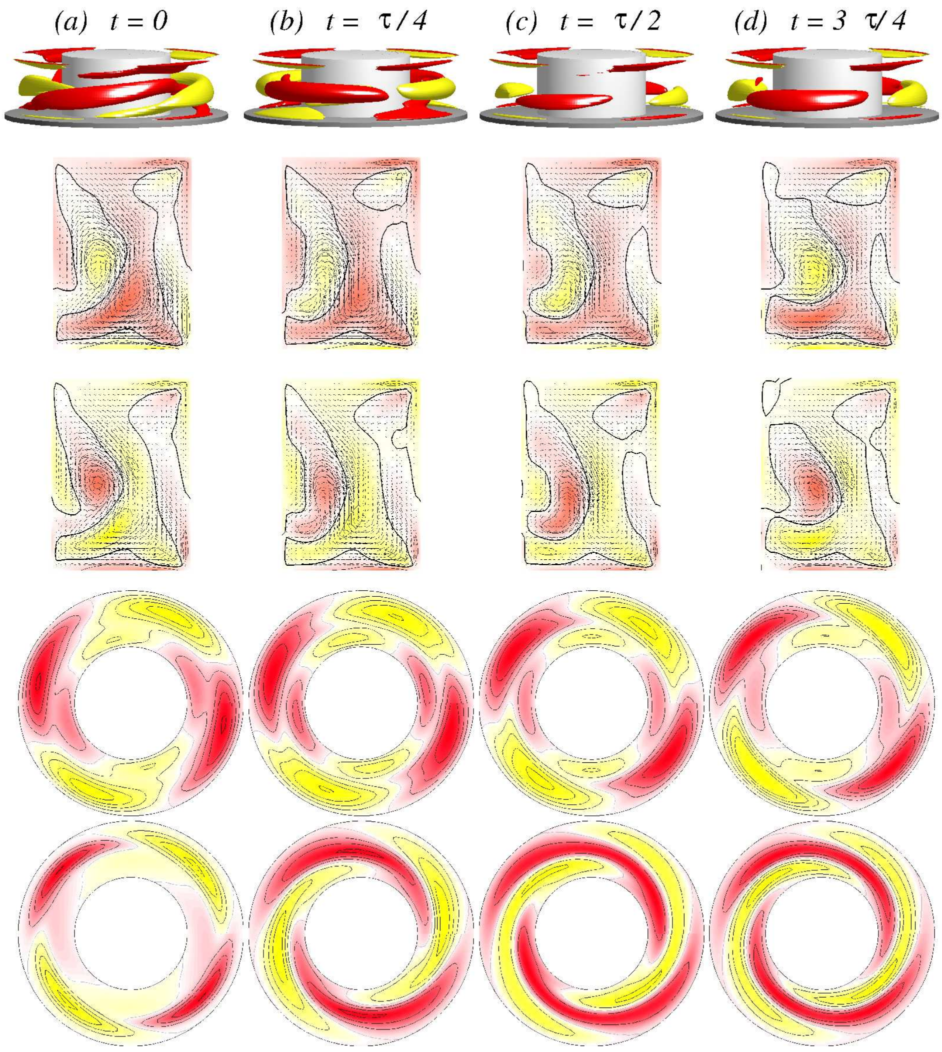

FIG. 7: **Visualization of the azimuthally oscillating twin-cell flow state** $2T_2^{\theta\text{-osci}}$ for $\Gamma = 1.2$ and $Re_2 = -500$. Legends are the same as in Fig. 4. Top row shows the isosurfaces for $rv = \pm 15$. The period is $\tau_\theta \approx 0.09859$. See movie files movieC1.avi, movieC2.avi and movieC3.avi.

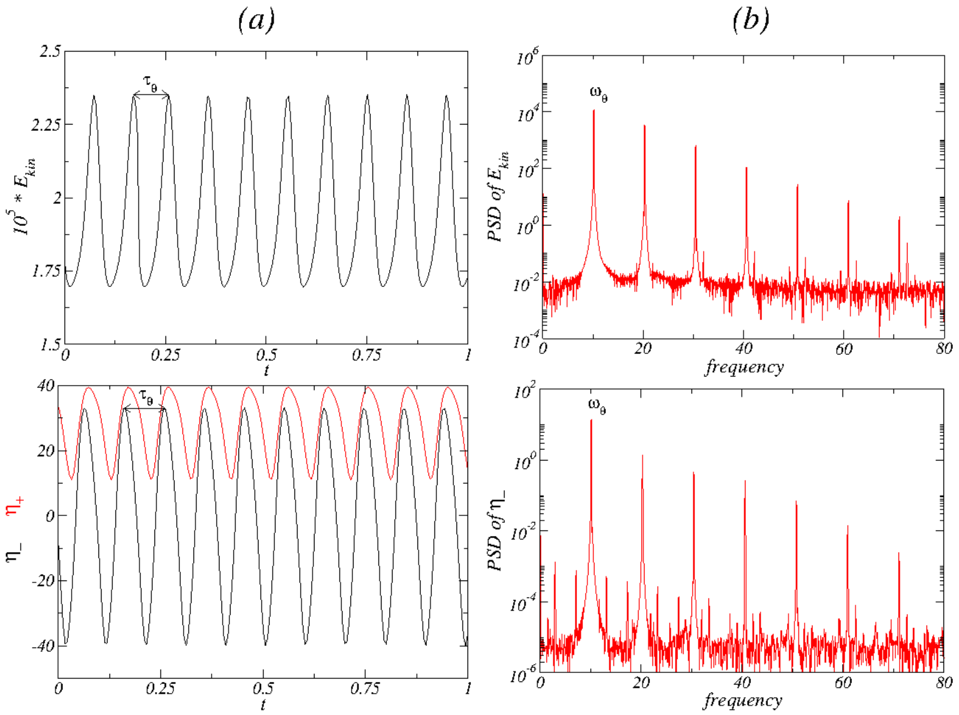

FIG. 8: **Time series and PSDs for azimuthally oscillating twin-cell flow** $2T_2^{\theta\text{-osci}}$ for $\Gamma = 1.2$ and $Re_2 = -500$. (a) Time series of E_{kin}, η_+ [red (gray)], and η_- (black). (b) The corresponding PSDs. The period of axial oscillation is $\tau_\theta \approx 0.09859$ with the corresponding frequency $\omega_\theta \approx 10.143$.

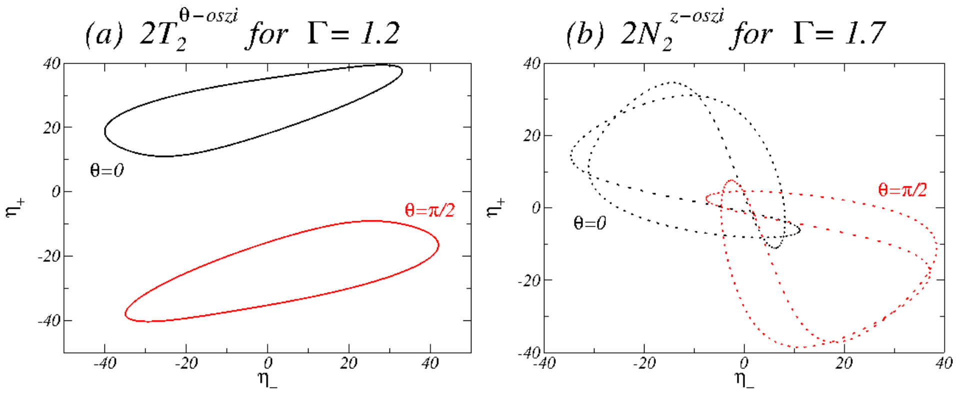

FIG. 9: **Phase portraits of flow states for** $Re_2 = -500$**.** Phase portraits of (a) $2T_2^{\theta\text{-osci}}$ for $\Gamma = 1.2$ and (b) $2N_2^{z\text{-osci}}$ for $\Gamma = 1.7$ on the (η_+, η_-) plane. Black [red (gray)] curves correspond to the azimuthal position $\theta = 0$ [$\theta = \pi/2$]. The partially filled cycle for $2N_2^{z\text{-osci}}$ results from the extremely long simulation time required.

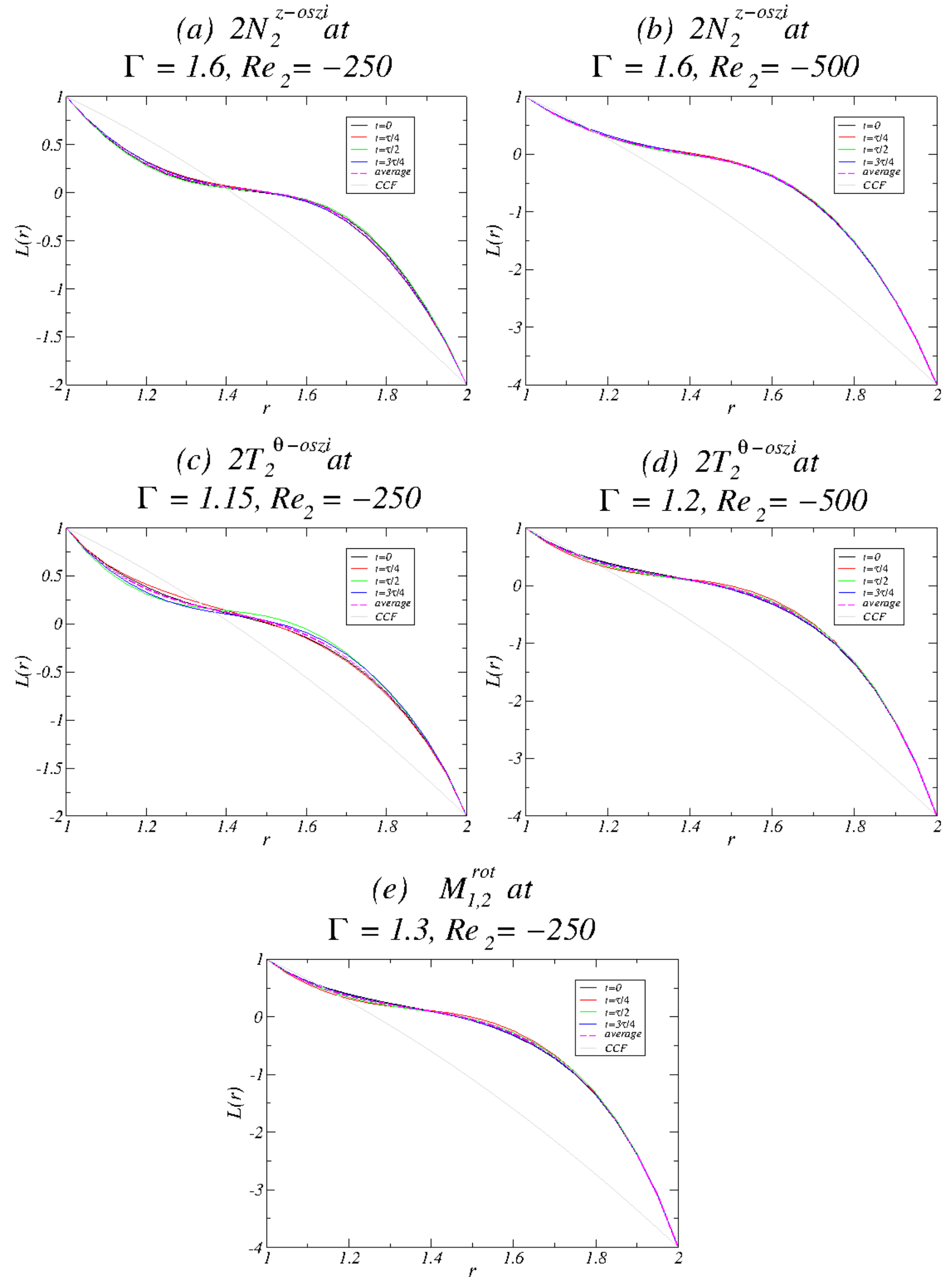

FIG. 10: **Behavior of the angular momentum with Γ.** Normalized angular momentum $L(r) = r\langle v(r)\rangle_{\theta,z}/Re_1$ versus the radius r for values of Re_2 and Γ as indicated. Dashed curves are the time-averaged values for the unsteady flow states. The gray thin solid line specifies the case for the unstable equilibrium circular Couette flow (CCF).

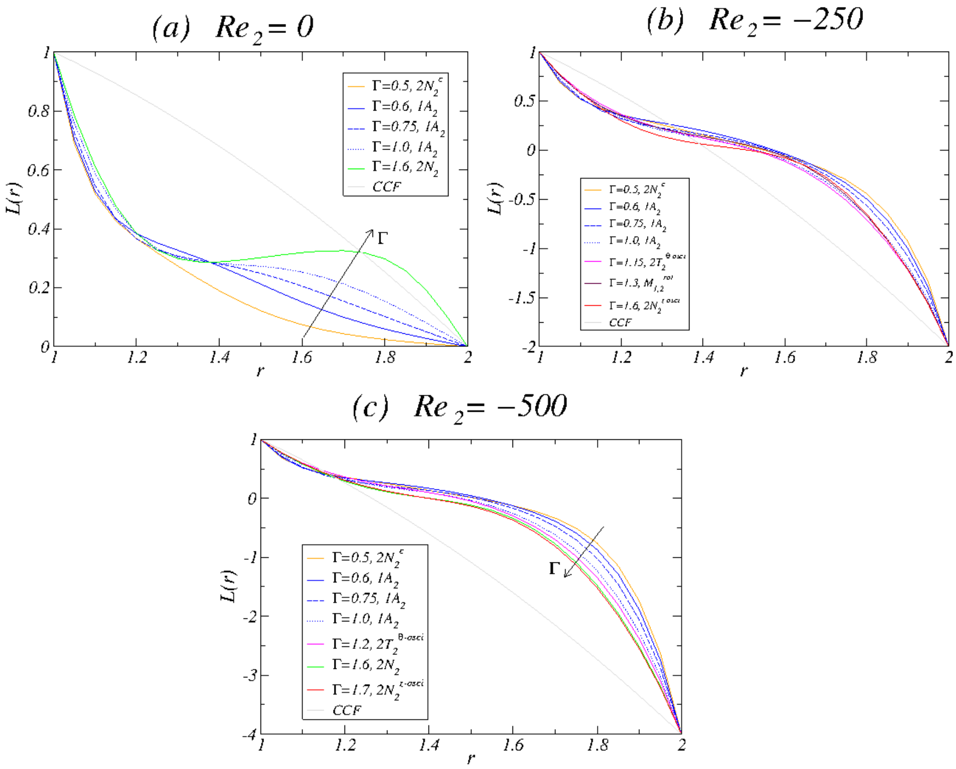

FIG. 11: **Behavior of the angular momentum for unsteady flow states.** Legends are the same as for Fig. 10. Dashed curves indicate the (one period) averaged values. Variations in the angular momentum over one period are quite small for axially oscillating flow states, but are moderately large for azimuthally oscillating or rotating flow states.

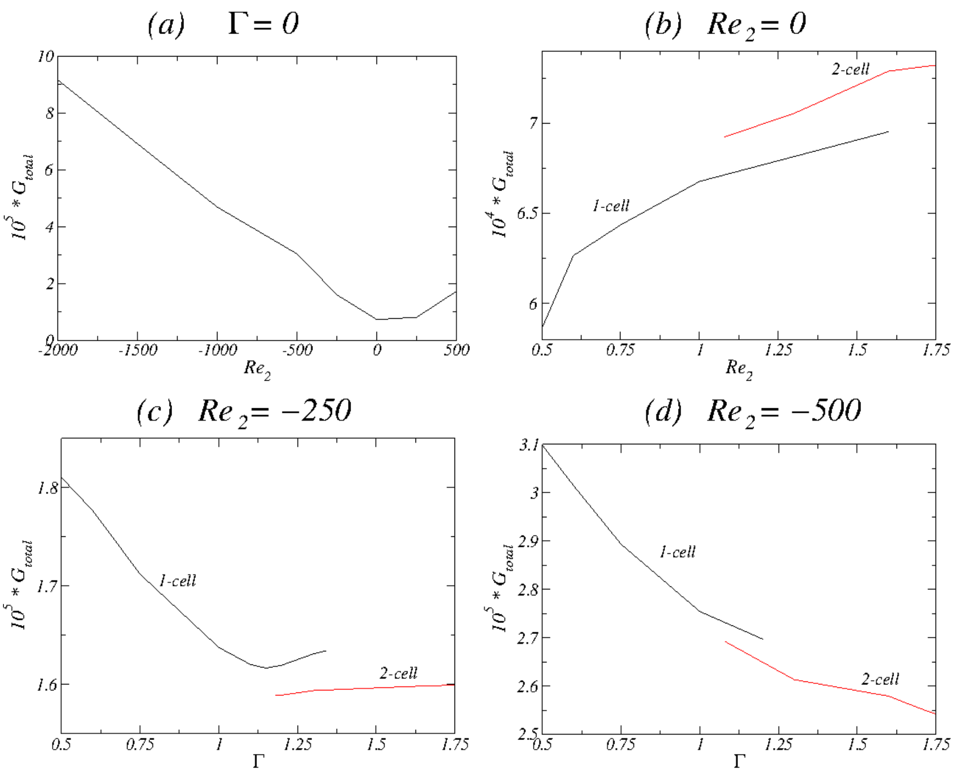

FIG. 12: **Behaviors of torque.** For various flow states at the values of parameters as indicated, the dimensionless torque $G = \nu J^{\omega}$ versus (a) Re_2 and $(b-d)$ Γ. See text for details.

Legends for videos in SM

- MovieA1:
MovieA1 demonstrates the *axially oscillating* two-cell flow state $2N_2^{\text{z-osci}}$, isosurfaces of the angular momentum $rv = \pm 15$ (red: $rv = 15$, yellow: $rv = -15$). Period time $\tau_z \approx 0.1635$; further parameters are $\Gamma = 1.6$ and $Re_2 = -250$.

- MovieA2:
MovieA2 demonstrates the *axially oscillating* two-cell flow state $2N_2^{\text{z-osci}}$, azimuthal velocity $v(r, \theta)$ in the axial planes $z = \Gamma/2$ at mid-hight (red and yellow colors correspond to positive and negative values, respectively, with zero specified as white). Period time $\tau_z \approx 0.1635$; further parameters are $\Gamma = 1.6$ and $Re_2 = -250$.

- MovieA3:
MovieA3 demonstrates the *axially oscillating* two-cell flow state $2N_2^{\text{z-osci}}$, vector plots $[u(r, z), w(r, z)]$ of the radial and axial velocity components in the planes defined by $\theta = 0$ with the color-coded azimuthal vorticity field η (red: $\eta > 0$, yellow: $\eta < 0$). Period time $\tau_z \approx 0.1635$; further parameters are $\Gamma = 1.6$ and $Re_2 = -250$.

- MovieA4:
MovieA4 demonstrates the *axially oscillating* two-cell flow state $2N_2^{\text{z-osci}}$, vector plots $[u(r, z), w(r, z)]$ of the radial and axial velocity components in the planes defined by $\theta = \pi/2$ with the color-coded azimuthal vorticity field η (red: $\eta > 0$, yellow: $\eta < 0$). Period time $\tau_z \approx 0.1635$; further parameters are $\Gamma = 1.6$ and $Re_2 = -250$.

- MovieB1:
MovieB1 demonstrates the *azimuthally oscillating* twin-cell flow state $2T_2^{\theta\text{-osci}}$, isosurfaces of the angular momentum $rv = \pm 25$ (red: $rv = 25$, yellow: $rv = -25$). Period time $\tau_\theta \approx 0.0954$; further parameters are $\Gamma = 1.15$ and $Re_2 = -250$.

- MovieB2:
MovieB2 demonstrates the *azimuthally oscillating* twin-cell flow state $2T_2^{\theta\text{-osci}}$, vector plots $[u(r, z), w(r, z)]$ of the radial and axial velocity components in the planes defined by $\theta = 0$ with the color-coded azimuthal vorticity field η (red: $\eta > 0$, yellow: $\eta < 0$). Period time $\tau_\theta \approx 0.0954$; further parameters are $\Gamma = 1.15$ and $Re_2 = -250$.

- MovieB3:
MovieB3 demonstrates the *azimuthally oscillating* twin-cell flow state $2T_2^{\theta\text{-osci}}$, vector plots $[u(r, z), w(r, z)]$ of the radial and axial velocity components in the planes defined by $\theta = \pi/2$ with the color-coded azimuthal vorticity field η (red: $\eta > 0$, yellow: $\eta < 0$). Period time $\tau_\theta \approx 0.0954$; further parameters are $\Gamma = 1.2$ and $Re_2 = -250$.

- MovieB4:
MovieB4 demonstrates the *axially oscillating* two-cell flow state $2T_2^{\text{z-osci}}$, azimuthal velocity $v(r, \theta)$ in the axial planes $z = \Gamma/2$ at mid-hight (red and yellow colors correspond to positive and negative values, respectively, with zero specified as white). Period time $\tau_\theta \approx 0.0954$; further parameters are $\Gamma = 1.2$ and $Re_2 = -250$.

- MovieC1:
MovieC1 demonstrates the *azimuthally oscillating* twin-cell flow state $2T_2^{\theta\text{-osci}}$, isosurfaces of the angular momentum $rv = \pm 15$ (red: $rv = 15$, yellow: $rv = -15$). Period time $\tau_\theta \approx 0.09859$; further parameters are $\Gamma = 1.15$ and $Re_2 = -250$.

- MovieC2:
MovieC2 demonstrates the *azimuthally oscillating* twin-cell flow state $2T_2^{\theta\text{-osci}}$, vector plots $[u(r, z), w(r, z)]$ of the radial and axial velocity components in the planes defined by $\theta = 0$ with the color-coded azimuthal vorticity field η (red: $\eta > 0$, yellow: $\eta < 0$). Period time $\tau_\theta \approx 0.09859$; further parameters are $\Gamma = 1.2$ and $Re_2 = -250$.

- MovieC3:
MovieC3 demonstrates the *azimuthally oscillating* twin-cell flow state $2T_2^{\theta\text{-osci}}$, vector plots $[u(r, z), w(r, z)]$ of the radial and axial velocity components in the planes defined by $\theta = \pi/2$ with the color-coded azimuthal vorticity field η (red: $\eta > 0$, yellow: $\eta < 0$). Period time $\tau_\theta \approx 0.09859$; further parameters are $\Gamma = 1.2$ and $Re_2 = -250$.

- MovieD1:
MovieD1 demonstrates the *rotating* flow state $M_{1,2}^{\text{rot}}$, isosurfaces of the angular momentum $rv = \pm 25$ (red: $rv = 25$, yellow: $rv = -25$). Period time $\tau_{rot} \approx 0.7829$; further parameters are $\Gamma = 1.3$ and $Re_2 = -250$.

- MovieD2:
MovieD2 demonstrates the *rotating* flow state $M_{1,2}^{\text{rot}}$,, azimuthal velocity $v(r,\theta)$ in the axial planes $z = \Gamma/2$ at mid-hight (red and yellow colors correspond to positive and negative values, respectively, with zero specified as white). Period time $\tau_{rot} \approx 0.7829$; further parameters are $\Gamma = 1.3$ and $Re_2 = -250$.

- MovieE1:
MovieE1 demonstrates the *azimuthally oscillating* twin-cell flow state $2T_2^{\text{z-osci}}$, isosurfaces of the angular momentum $rv = \pm 15$ (red: $rv = 15$, yellow: $rv = -15$). Period time $\tau_z \approx 0.157$; further parameters are $\Gamma = 1.7$ and $Re_2 = -500$.

- MovieE2:
MovieE2 demonstrates the *azimuthally oscillating* twin-cell flow state $2T_2^{\text{z-osci}}$, vector plots $[u(r,z), w(r,z)]$ of the radial and axial velocity components in the planes defined by $\theta = 0$ with the color-coded azimuthal vorticity field η (red: $\eta > 0$, yellow: $\eta < 0$). Period time $\tau_z \approx 0.157$; further parameters are $\Gamma = 1.7$ and $Re_2 = -500$.

- MovieE3:
MovieE3 demonstrates the *azimuthally oscillating* twin-cell flow state $2T_2^{\text{z-osci}}$, vector plots $[u(r,z), w(r,z)]$ of the radial and axial velocity components in the planes defined by $\theta = \pi/2$ with the color-coded azimuthal vorticity field η (red: $\eta > 0$, yellow: $\eta < 0$). Period time $\tau_z \approx 0.157$; further parameters are $\Gamma = 1.7$ and $Re_2 = -500$.

---

* Electronic address: sebastian.altmeyer@ist.ac.at
† Electronic address: yhdo@knu.ac.kr
[1] Eckhardt, B. & Grossmann, S. & Lohse, D. Torque scaling in turbulent Taylor-Couette flow between independently rotating cylinders. *J. Fluid Mech.* **581**, 221–250 (2007).
